# Liver ubiquitome uncovers nutrient-stress-mediated trafficking and secretion of complement C3

**DOI:** 10.1038/cddis.2016.312

**Published:** 2016-10-13

**Authors:** Helena de Fatima Magliarelli, Mariette Matondo, Gergő Mészáros, Alexander Goginashvili, Eric Erbs, Zhirong Zhang, Michael Mihlan, Christian Wolfrum, Ruedi Aebersold, Izabela Sumara, Romeo Ricci

**Affiliations:** 1 Institut de Génétique et de Biologie Moléculaire et Cellulaire (IGBMC), Centre National de la Recherche Scientifique UMR 7104, Institut National de la Santé et de la Recherche Médicale U964, Université de Strasbourg, Illkirch 67404, France; 2 Department of Biology, Institute of Molecular Systems Biology, Eidgenössische Technische Hochschule (ETH), Zurich, Switzerland; 3 Nouvel Hôpital Civil, Laboratoire de Biochimie et de Biologie Moléculaire, Université de Strasbourg, Strasbourg, France; 4 Department of Health Sciences and Technology, ETH Zurich, Zurich, Switzerland; 5 Faculty of Science, University of Zurich, Zurich, Switzerland

## Abstract

Adaptation to changes in nutrient availability is crucial for cells and organisms. Posttranslational modifications of signaling proteins are very dynamic and are therefore key to promptly respond to nutrient deprivation or overload. Herein we screened for ubiquitylation of proteins in the livers of fasted and refed mice using a comprehensive systemic proteomic approach. Among 1641 identified proteins, 117 were differentially ubiquitylated upon fasting or refeeding. Endoplasmic reticulum (ER) and secretory proteins were enriched in the livers of refed mice in part owing to an ER-stress-mediated response engaging retro-translocation and ubiquitylation of proteins from the ER. Complement C3, an innate immune factor, emerged as the most prominent ER-related hit of our screen. Accordingly, we found that secretion of C3 from the liver and primary hepatocytes as well as its dynamic trafficking are nutrient dependent. Finally, obese mice with a chronic nutrient overload show constitutive trafficking of C3 in the livers despite acute changes in nutrition, which goes in line with increased C3 levels and low-grade inflammation reported for obese patients. Our study thus suggests that nutrient sensing in the liver is coupled to release of C3 and potentially its metabolic and inflammatory functions.

In vertebrates, the liver represents a metabolic hub allowing complex organisms to cope with nutritional challenges: keeping glucose levels steady in the blood during fasting, and storing excess of nutrients in the form of glycogen and lipids in the postprandial state.^1, 2^ At the cellular level, hepatocytes engage a metabolic stress program *in situations* of insufficient supply of nutrients, as well as during nutrient excess.^3^ During low-nutrient conditions, glucose and amino-acid deficiency induce, among other responses, autophagy to mitigate cellular damage and provide nutrients for short-term survival.^4^ On the other hand, nutrient excess can alter mitochondrial function and endoplasmic reticulum (ER) metabolism, resulting in increased ROS production culminating in the activation of stress kinases, including c-jun N-terminal kinases (JNKs), I*κ*B kinase, p38 and double-stranded RNA-dependent kinase.^3^ Fructose represents one important factor that induces cellular stress in hepatocytes. The metabolism of fructose by hepatocytes leads to specific activation of JNK and is strongly associated with hepatic insulin resistance as well as induction of *de novo* lipogenesis increasing intracellular lipid content.^5, 6^ The consumption of fructose has increased dramatically over the past decades and is being linked to the development of insulin resistance, hypertriglyceridemia, type 2 diabetes and non-alcoholic fatty liver disease.^7, 8^

Nutritional inputs induce a complex array of changes in posttranslational modifications (PTMs) contributing to adaptation of cellular functions to metabolic stress. Ubiquitin conjugation (ubiquitylation) to substrate proteins remains relatively poorly explored in a metabolic context. Regulation of hepatic glucose production represents one critical metabolic process regulated through ubiquitylation.^9, 10, 11, 12^ Ubiquitylation is a versatile PTM that was initially discovered to be a mark for targeting proteins to proteasomal degradation.^13^ However, ubiquitin is now considered as a signaling tag able to trigger a whole plethora of molecular events in cells.^14, 15^ Protein ubiquitylation can exist in many different topologies: from a single ubiquitin molecule linked to substrates to a large variety of polyubiquitin chains.^14, 15^ While ubiquitin-binding domain proteins recognize these different ubiquitin topologies and determine the fate of substrates in the cell and the deubiquitylating enzymes reverse ubiquitylation or control editing of polyubiquitin chains.^14, 16, 17^

Herein we aimed at uncovering ubiquitylation pathways regulated in a nutrient-responsive manner and their role during a nutrient-stress response. To this end, we performed a proteomic screen for ubiquitylated proteins in the livers of mice subjected to nutritional stress conditions imposed by switching from fasting to refeeding with a high sucrose diet. We provide a comprehensive list of differentially ubiquitylated proteins linking ubiquitylation events to metabolic processes in the liver. Moreover, we studied one of the strongest hits of our screen, complement C3 that is produced and secreted by the liver to act as a major component of innate immune defense, unraveling that trafficking and secretion of complement C3 was nutrient dependent. We hypothesize that nutrient sensing in the liver is coupled to release of C3 and potentially its metabolic and inflammatory functions.

## Results

### Identification of the ubiquitin-modified proteome in response to nutrient stress in the liver

**Figure 1:**
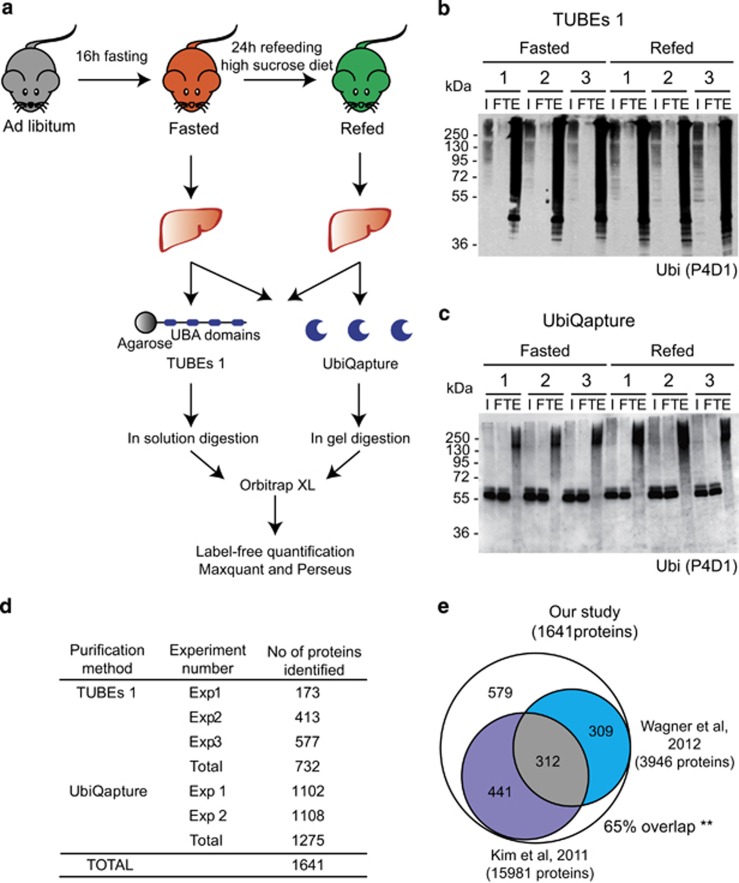
Identification of the ubiquitin-modified proteome in response to nutrient stress in the liver. (**a**) Schematic representation of each step of the proteomic approach. Livers of 16 h fasted mice (in orange) and 24 h high-sucrose refed mice (in green) were isolated, and total liver extracts were purified with both TUBEs 1 and UbiQapture followed by in-solution or in-gel trypsin digestion, respectively. Samples were subjected to an Orbitrap XL and analyzed by Maxquant and Perseus.^46^ (**b** and **c**) Western blottings with samples of TUBEs 1- (**b**) and UbiQapture-purified (**c**) liver lysates of three fasted and three refed mice. An antibody against ubiquitin (P4D1) was used to detect ubiquitylated proteins. Input (I), flow through (FT) and elution (E) are shown. (**d**) Table with numbers of identified proteins of independent experiments. Three independent experiments were performed with TUBEs 1 pulldowns with lysates from 26 mice in total. Two independent experiments were performed with UbiQapture pulldowns with lysates from 20 mice in total. Total numbers of unique proteins are indicated for both approaches. Merging of both numbers resulted in identification of 1641 unique proteins. (**e**) Venn diagrams comparing proteins identified in our screen with two previous studies,^20, 21^ respectively. Five hundred and seventy-nine proteins (65% of all identified proteins) in our screen overlapped with proteins identified in these studies. **P<0.01

To identify proteins that are ubiquitylated in response to a nutritional stress in the mouse liver, we performed a fasting–refeeding experiment (Figure 1a). The high sucrose diet is used to trigger a strong induction of *de novo* lipogenesis in the liver^18^ associated with high cellular stress.^7^ Indeed, expression of genes involved in gluconeogenesis and fatty acid oxidation was increased during fasting, while one of the glycolytic and lipogenic genes was elevated during refeeding (Supplementary Figures S1A–D).

Two complementary approaches were used to pulldown ubiquitylated proteins from liver lysates: Tandem Ubiquitin-Binding Entities 1 (TUBEs 1)^19^ and UbiQapture (Figures 1b and c). Both approaches allowed for efficient purification of ubiquitylated proteins from total liver extracts (Figures 1b and c) and HEK293T cells (Supplementary Figures S2A and B). For example, we observed enriched ubiquitylation of I*κ*B*α* in response to tumor necrosis factor *α* (TNF*α*) upon inhibition of proteasomal degradation (Supplementary Figures S2A and B). We thus next used TUBEs 1 and UbiQapture to purify potentially ubiquitylated proteins from liver lysates of mice subjected to fasting–refeeding prior to mass spectrometric analysis (Figures 1b and c).

Mass spectrometric analysis revealed 1641 unique putative ubiquitylated proteins (Figure 1d and Supplementary Table S1). Importantly, 65% of the identified proteins were previously shown to be directly ubiquitylated in two large-scale proteomic studies^20, 21^ (Figure 1e), confirming the relevance of our approach. A hypergeometric distribution analysis showed that these overlaps were statistically significant and non-random. There was also a significant overlap between experimental replicates: 50% of proteins were identified in at least two experiments with TUBEs 1 (Supplementary Figure S3A) and 73% of proteins were found in both experiments with UbiQapture (Supplementary Figure S3B). In addition, 22% of the proteins were detected with both techniques (Supplementary Figure S3C). These data thus suggest that some of the proteins were recognized by both ubiquitin-binding entities, but most of the substrates had different binding affinities.

### Fasting–feeding alters ubiquitylation patterns in the liver

**Figure 2:**
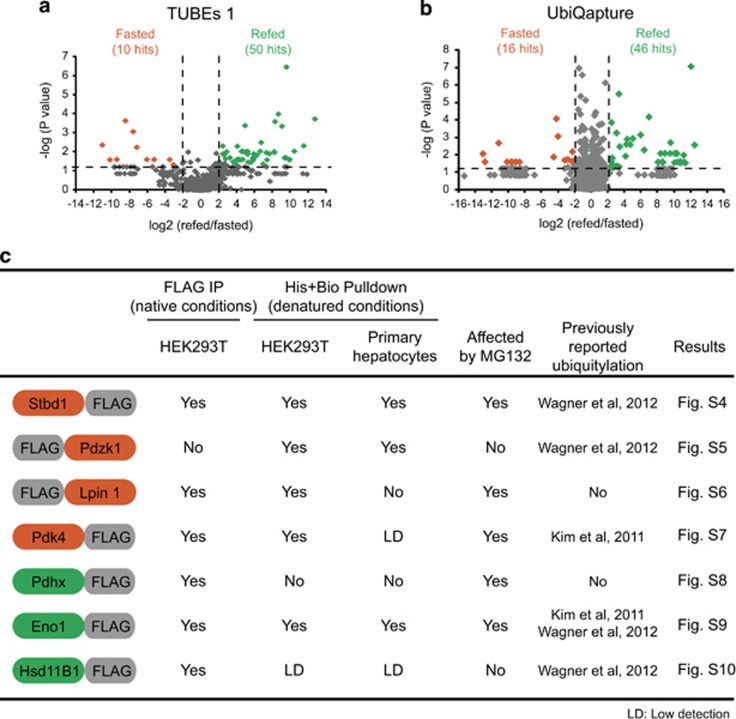
Fasting–feeding alters ubiqitylation patterns in the liver. (**a** and **b**) Volcano plots representing all identified proteins classified according to log2 of the difference between refeeding and fasting (*x* axis) and the negative log of the *P*-values obtained by ANOVA (*y* axis). Thresholds of four-fold differences between fasted and refed and *P*-values <0.05 (dotted lines) were chosen to select putatively ubiquitylated proteins in the fasted (orange) and refed livers (green). Number of hits are shown separately for TUBEs 1- (**a**) and UbiQapture-purified (**b**) proteins. (**c**) Summary of all the IPs and pulldowns performed to confirm the ubiquitylation of seven proteins selected for biochemical validation. ‘Yes' refers to ubiquitylation identified in at least one of the conditions tested; ‘No' refers to ubiquitylation not identified in the conditions tested. Flag tag was added to either N- or C-terminus depending on the domains present in the protein. Proteins not detected in the input were assigned with LD (low detection)

To identify proteins that were significantly more abundant in ubiquitin purifications under fasting or refeeding conditions, we used label-free quantification and performed one-way analysis of variance (ANOVA), which served to build the volcano plots (Figures 2a and b). We identified 26 proteins in the livers of fasted (Supplementary Table S2) and 91 proteins in the livers of refed mice (Supplementary Table S3). To rigorously validate our proteomic approach, we selected seven hits representing genes implicated in metabolism (*Stbd1*, *Pdzk1*, *Lpin1*, *Pdk4*, *Pdhx*, *Eno1*, *Hsd11B1*). To confirm their ubiquitylation, we used HEK293T cells and primary murine hepatocytes (Supplementary Figure S3D). We could demonstrate ubiquitylation of all seven candidate proteins (Figure 2c and Supplementary Figures S4–S10) further confirming the relevance of our proteomic analysis.

**Figure 3:**
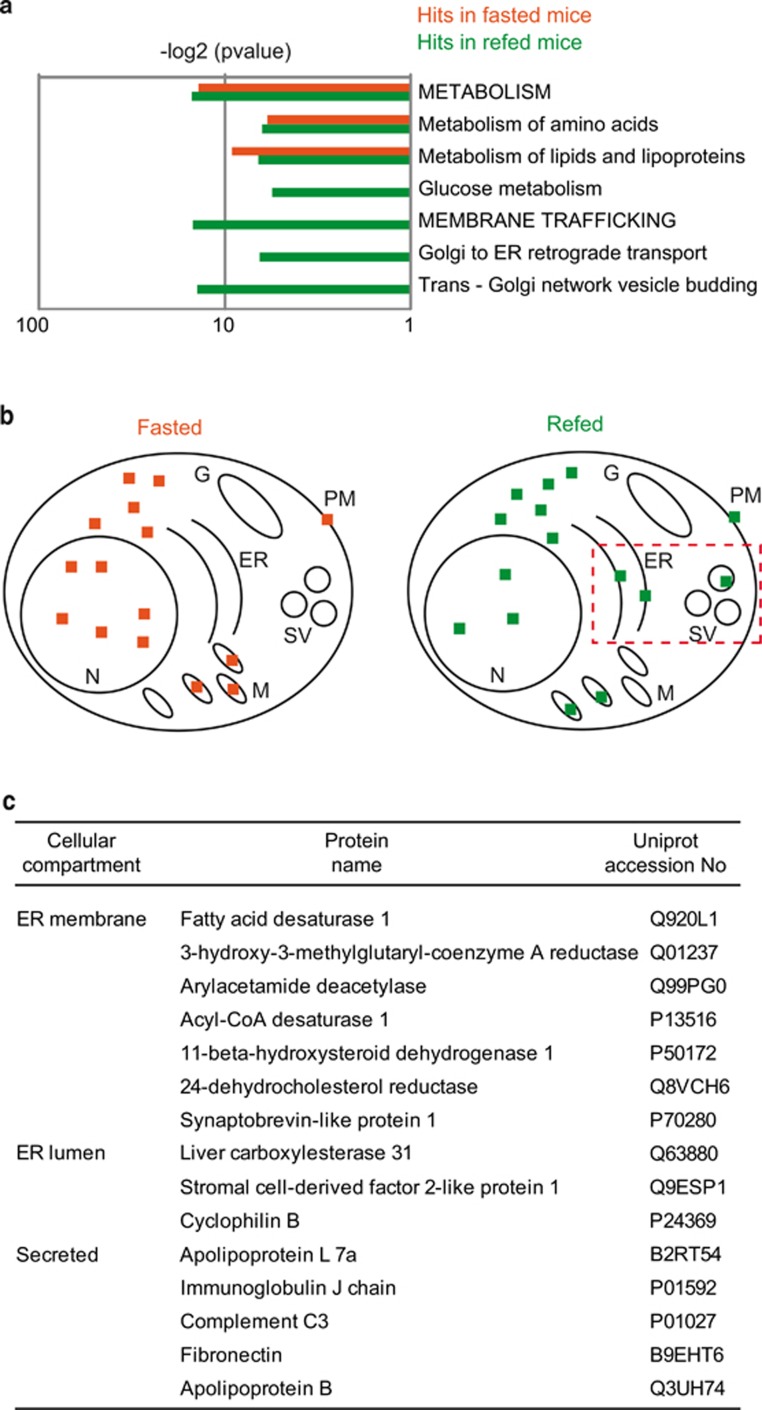
Liver ubiquitome identifies ER-resident and secretory proteins. (**a**) Bar chart depicting the significance (*P*-value) for enriched indicated pathways/processes. Values for significantly enriched proteins in respective pathways/processes detected in the fasted (orange) or refed (green) livers. Only a selected group of pathways/processes is shown. A complete analysis is provided in Supplementary Figure S11C. −Log2 of *P*-values were used to indicate hits in fasted *versus* refed mice. (**b**) Graphical representation of differentially ubiquitylated proteins with respect to their localization in the indicated cellular compartments. Following compartments have been assigned to candidate proteins according to UniProt: nucleus (N), mitochondria (M), ER, Golgi (G) and secretory vesicles (SV), and plasma membrane (PM). Number of dots indicate the relative percentage of proteins localized to respective compartments (6.25% per dot, 16 dots in total). Proteins with unknown localization corresponded to 1 dot in both conditions. Red dotted rectangle highlights enrichment of hits at the ER and SV. (**c**) Table with cellular localization, protein name and UniProt number of all ER-resident and secreted proteins identified in refed mice

Gene ontology analysis revealed significant clustering of identified proteins predominantly localized to mitochondria, within ribonucleoprotein complexes and to ER (Supplementary Figures S11A and B). Moreover, functional clustering showed significant enrichment of metabolic pathways such as metabolism of amino acids and lipids under both fasting and refeeding conditions (Figure 3a). Interestingly, among the identified hits, proteins involved in membrane trafficking were enriched upon refeeding of mice (Figures 3b and c and Supplementary Figure S11C). Indeed, under nutrient-stress conditions, the liver is responsible for secreting proteins, such as very low-density lipoproteins.^22^ In line with this notion, we observed that 16% of the proteins identified in refed mice were either secreted, ER membrane or luminal proteins in contrast to proteins identified in fasted mice, which were predominantly located in the cytosol, mitochondria and nucleus (Figures 3b and c). Thus our findings suggest that adaptation of the secretory machinery to nutrient availability is critical in the liver and might be linked to ubiquitin signaling.

### Pro-C3 is ubiquitylated in the livers of refed mice

**Figure 4:**
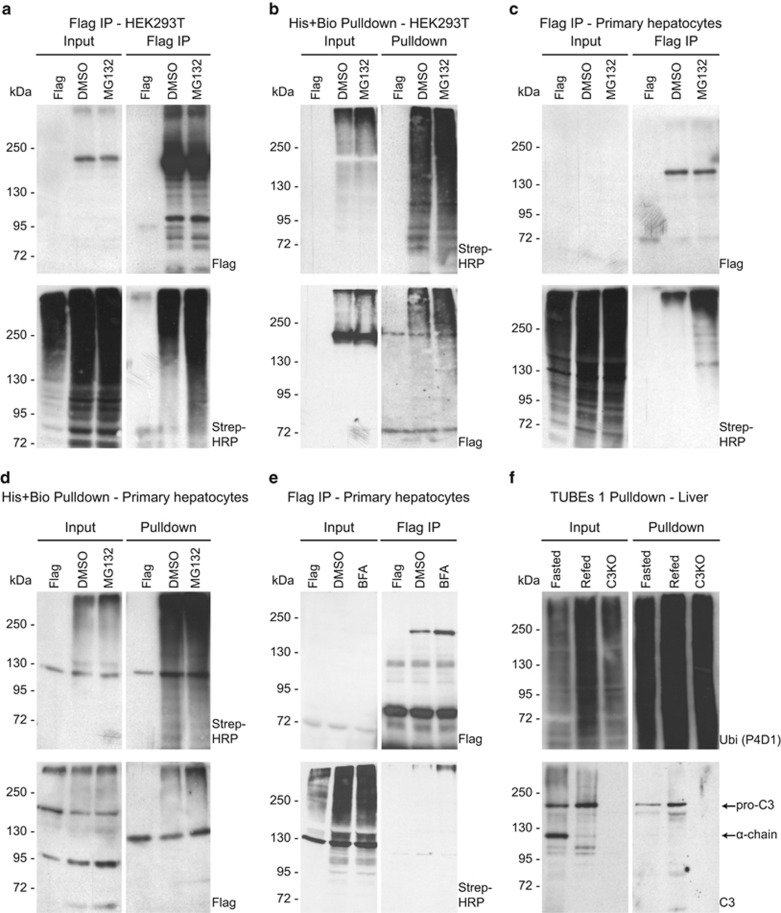
Pro-C3 is ubiquitylated in the livers of refed mice. (**a** and **b**) Western blotting after anti-FLAG immunoprecipitation (FLAG IP) (**a**) and histidin-biotin pulldowns (His-Bio pulldown) (**b**) of whole-cell lysates from HEK293T cells ectopically expressing FLAG-tagged C3 and His-Bio-tagged ubiquitin. Cells expressing FLAG alone were used as a control. Cells were treated with DMSO, 10 *μ*M of proteasome inhibitor MG132 or 50 *μ*g/ml BFA for 4 h as indicated. Input, FLAG IP or HIS-Bio pulldowns are shown separately. Signals with an antibody against FLAG-tag or revealed by streptavidin conjugated to horseradish peroxidase (Strep-HRP) are shown separately. (**c**–**e**) Western blotting after FLAG-IP (**c** and **e**) and a His-Bio pulldown (**d**) of whole-cell lysates from primary murine hepatocytes ectopically expressing FLAG-tagged C3 and His-Bio-tagged ubiquitin. Cells expressing FLAG alone were used as a control. Cells were treated with DMSO, MG132 (**c** and **d**) or BFA (**e**) as indicated. Input, FLAG IP or HIS-Bio pulldowns are shown separately. Signals with an antibody against FLAG tag or revealed by streptavidin conjugated to horseradish peroxidase (Strep-HRP) are shown separately. (**f**) Western blotting after TUBEs 1 pulldowns of whole-liver lysates from fasted and refed mice as indicated. Input and pulldowns are shown separately. Liver lysates from C3 knockout mice (C3KO) were used as a control. Antibodies against ubiquitin (P4D1) and C3a were used. Arrows indicate endogenous pro-C3 and the alpha chain (*α* chain) of mature C3

ER-stress response is a cellular adaptation process triggered by a variety of conditions, including nutrient overload.^23^ In our experimental model, high sucrose diet induced the expression of ER-stress markers (Supplementary Figure S12A) and the phosphorylation of inositol-requiring enzyme 1 alpha (p-IRE1*α*), the classical marker of unfolded protein response (Supplementary Figure S12B). Furthermore, our screening results suggest that ER-resident and secreted proteins are differentially ubiquitylated upon fasting and feeding conditions (Figure 3). Among the most prominent hits, we identified complement C3, a central component of the innate immunity (Supplementary Table S3, Figure 3c). In addition to its canonical immunological functions, complement C3 is known to have a role in metabolism by increasing lipogenesis in adipose tissue and enhancing insulin secretion.^24, 25, 26^ Moreover, elevated blood levels of complement C3 were reported in obese and diabetic patients.^27, 28, 29^ Thus nutrient-dependent ubiquitylation of complement C3 might be linked to its trafficking and secretion. To test this hypothesis, ubiquitylation of complement C3 was tested in HEK293T cells and in primary murine hepatocytes (Figures 4a–d). Strikingly, we observed that complement C3 was ubiquitylated using all different experimental conditions (Figures 4a–d). Moreover, treatment with the proteasome inhibitor MG132 increased the amount of ubiquitylated C3 in HEK293 cells (Figures 4a and b) and in primary hepatocytes (Figures 4c and d), suggesting that ubiquitylated C3 was at least partially subjected to proteasomal degradation.

**Figure 5:**
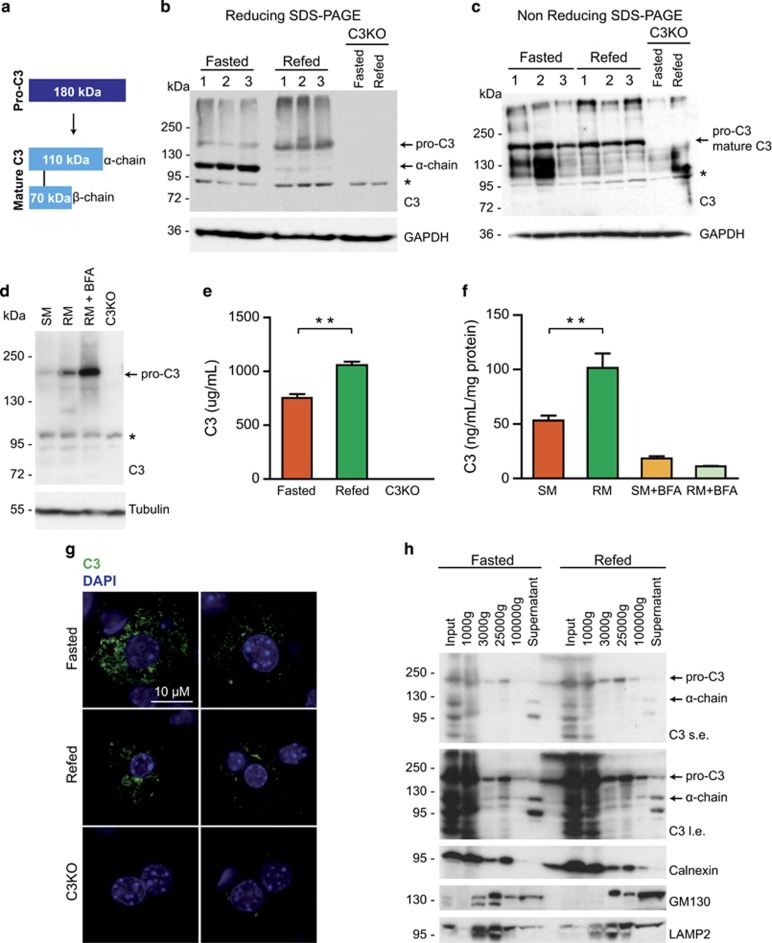
Mature C3 is stored in small vesicles and secreted upon nutrient overload. (**a**) Schematic representation of processing of pro-C3 to mature C3. A disulfide bond covalently links the indicated alpha and *β*-chain of mature C3. (**b** and **c**) Western blotting of whole-liver lysates from fasted and refed mice. Proteins were separated by SDS-page under reducing (**b**) and non-reducing (**c**) conditions. An antibody against C3a was used. Arrows indicate endogenous pro-C3 and the alpha chain (*α* chain) of mature C3 in panel (**b**) or pro-C3/mature C3 in panel (**c**). Asterisk (*) indicate unspecific band. GAPDH was used as a loading control. Liver lysates from C3 knockout mice (C3KO) were used as negative controls. (**d**) Western blotting of whole-cell lysates from primary murine hepatocytes subjected to starvation (SM) or nutrient-rich media (RM) without or with 50 *μ*g/ml BFA. C3KO lysates were used as a control. An antibody against C3a was used. The arrow indicates endogenous pro-C3. Asterisk (*) indicate unspecific band. Tubulin was used as a loading control. (**e**) Quantification of total C3 in serum of fasted and refed mice by enzyme-linked immunosorbent assay (ELISA). Sera from C3KO mice were used as a negative control. Data are presented as mean±S.E.M. of two individual experiments with serum from three different livers each. *P*<0.05. (**f**) Quantification of total C3 secreted from primary murine hepatocytes into cell culture supernatants by ELISA. Cells were subjected to starvation (SM) or nutrient-rich media (RM) without or with 50 *μ*g/ml BFA. Data are presented as means±S.E.M. of two individual experiments. *P*<0.05. (**g**) Intracellular localization of C3 in the livers of fasted and refed mice shown by immunofluorescence. Nuclei were stained with DAPI (4,6-diamidino-2-phenylindole). Livers of C3KO mice were used as a negative control. (**h**) Western blotting of whole-liver lysates after differential centrifugation from fasted and refed mice. Total lysates (Input), precipitates after 1000, 3000, 25 000 and 100 000 *g* as well as soluble fractions are shown. Antibodies against C3a, calnexin, GM130 and LAMP2 were used

To further test our hypothesis and functionally link complement C3 trafficking to ubiquitylation, we treated primary hepatocytes with Brefeldin A (BFA). Interestingly, we observed a moderate increase in the protein levels of complement C3 upon BFA treatment and increase in complement C3 ubiquitylation (Figure 4e), suggesting an involvement of ubiquitin signaling in trafficking of complement C3. To corroborate these findings, we have subsequently analyzed ubiquitylation of endogenous C3 in the livers of refed mice in which no proteasomal inhibition was applied (Figure 4f). C3 is first synthesized as pro-C3 in the ER as a single chain of 180 kDa. In the Golgi and in secretory vesicles, pro-C3 is processed into two chains linked by a disulfide bond (Figure 5a).^30^ Using an antibody specific for C3a, we detected two specific bands migrating at approximately 110 and 180 kDa in total liver lysates (Figure 4f). Lysates of the livers from C3 knockout mice confirmed specificity of these bands, which likely correspond to the *α*-chain of mature C3 and to pro-C3, respectively. Strikingly, while the upper band was more abundant in the livers of refed mice, the lower band was more abundant in the livers of fasted animals (Figure 4f). In TUBEs 1-purified lysates, a specific band at approximately 180 kDa, thus most likely corresponding to ubiquitylated pro-C3, was more abundant in refed mice, corroborating the data from the proteomic screen and higher levels of ubiquitylated C3 in refeeding conditions. We conclude that nutrient overload in the liver induces ubiquitylation of C3 and may control its intracellular trafficking and potentially secretion.

### Mature C3 is stored in small vesicles and secreted upon nutrient overload

In order to confirm that nutrient overload is linked to intracellular C3 trafficking, we first rigorously analyzed the identities of two bands detected in the livers with an anti-C3 antibody. To understand how the metabolic state of hepatocytes affected the abundance of these forms, we compared total lysates of the livers of fasted and refed mice under reducing and non-reducing SDS-PAGE conditions. Indeed, abundance of the two bands under reducing SDS-PAGE conditions changed in fasting and feeding, respectively, and the 180 kDa form was markedly increased upon feeding (Figure 5b). Importantly, under non-reducing conditions only the upper band was detected, strongly suggesting that the two bands indeed corresponded to pro-C3 and mature C3 (Figure 5c). The total C3 abundance was unchanged between the livers of fasted and refed mice (Figure 5c). This is in line with the fact that transcriptional levels of C3 were also not altered upon fasting and refeeding (Supplementary Figure S13A). A similar pattern of C3 processing was observed in primary hepatocytes cultured in starvation or in a nutrient-rich media and the abundance of a band migrating at around 180 kDa was increased upon nutrient addition (Figure 5d). In line with a previous report,^31^ we were unable to detect mature C3 in total hepatocyte extracts, which may indicate that isolated primary hepatocytes have a reduced capacity to store mature C3 prior to secretion. Expression of C3 at the RNA level was also not affected by our experimental conditions (Supplementary Figure S13B), suggesting that translation of pro-C3 might be enhanced under nutrient-rich conditions. Strikingly, treatment of primary hepatocytes kept in a nutrient-rich media with BFA, which is expected to prevent conversion from pro-C3 to mature C3, further increased abundance of the 180 kDa form of pro-C3, confirming our hypothesis that nutrient overload stimulates intracellular trafficking of C3 (Figure 5d). The lower levels of mature C3 observed in the livers of refed mice could be also explained by nutrient-dependent regulation of C3 secretion by hepatocytes. To test this possibility, we measured secreted C3 in the serum of fasted or refed mice and in the supernatant of cultured primary murine hepatocytes kept in a starvation or in a nutrient-rich media. Strikingly, we observed that levels of circulating C3 were higher in the serum of refed mice (Figure 4e), and hepatocytes subjected to a nutrient-rich media were secreting markedly increased levels of C3 as compared with those kept under starvation media (Figure 5f). As expected, BFA treatment markedly reduced secretion of C3 by hepatocytes (Figure 5f). Thus nutrient overload leads to increased secretion of C3 from hepatocytes.

To corroborate these findings, we next investigated the subcellular localization of C3 by immunofluorescence. C3 was found spread throughout the cytoplasm in the livers of both fasted and refed mice (Figure 5g). Interestingly, the C3 signal decreased dramatically in hepatocytes upon refeeding as compared with fasting. Although the intracellular C3 signal did not detectably localize to ER, Golgi or lysosomes (Supplementary Figures S13C–E), we observed moderately increased co-localization of C3 with these structures in the livers of fasted mice, indicating that C3 trafficking is slower under fasting conditions. A differential centrifugation assay using the liver extracts of fasted and refed mice confirmed these observations and showed that the majority of pro-C3 was present in fractions where ER and Golgi markers were identified and that the majority of the mature C3 was present in the soluble fraction (Figure 5h). Thus mature C3 might be stored in small Golgi-derived vesicles. Taken together, our data strongly suggest that mature C3 is rapidly released from hepatocytes in response to nutrients. Moreover, nutrient overload enhanced biogenesis of C3 most likely as a means to replenish C3 stores and to maintain secretion under feeding conditions.

### Acute and chronic nutritional stress accelerates trafficking of intracellular C3

**Figure 6:**
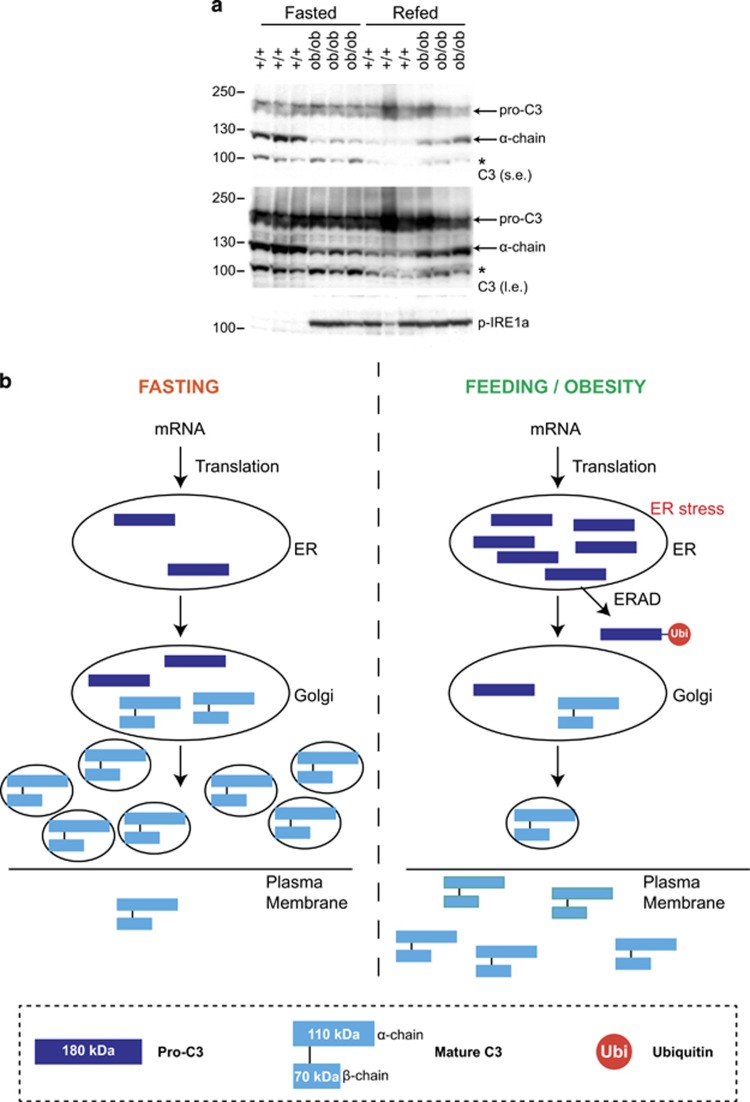
Acute and chronic nutritional stress accelerates trafficking of intracellular C3. (**a**) Western blotting of whole-liver lysates from wild-type and *ob/ob* fasted and refed mice. Proteins were separated by SDS-page under reducing conditions. An antibody against C3a was used. Short exposures (s.e.) and long exposures (l.e.) are shown. Arrows indicate endogenous pro-C3 and the alpha chain (*α* chain) of mature C3. Asterisk (*) indicate unspecific band. An antibody against p-IRE1*α* was used to assess ER stress. Liver lysates from C3 knockout mice (C3KO) were used as negative controls. (**b**) Model of C3 intracellular trafficking and secretion in conditions of fasting *versus* refeeding with high-sucrose diet or obesity

Our data show that acute nutrient overload in mice leads to ER-stress induction, ubiquitylation of pro-C3 and enhanced biogenesis and secretion of mature C3. Thus nutrient sensing could be coupled to metabolic and inflammatory functions of C3. It is intriguing that both increased C3 blood levels and low-grade inflammation were reported in obese patients.^27, 28, 29, 32, 33^ We therefore wondered whether a chronic metabolic challenge in mice would affect intracellular C3 trafficking. To this end, we measured C3 levels in hyperphagic and obese leptin-deficient (*ob/ob*) mice. Strikingly, in mouse liver samples we observed that mature C3 levels were decreased in *ob/ob* animals and in refed wild-type mice as compared with fasted control animals. The low levels of mature C3 strongly correlated with the high levels of ER stress in different metabolically challenged animals (Figure 6a). These results suggest that metabolic challenge in mice leads to increase in secretion of mature C3.

Taken together, our data suggest a model of regulation of intracellular C3 trafficking by nutrients. Under fasting conditions, pro-C3 is processed at the ER to the Golgi and the mature C3 form is stored in small secretory vesicles. Nutrient deprivation prevents C3 secretion to the blood. Nutrient-rich conditions stimulate intracellular trafficking of C3 and, as a consequence, its secretion. Nutrient overload may induce ER stress and expand the capacity of ER by both synthesis of chaperones and by ubiquitylation and degradation of misfolded fraction of secretory proteins. Under these conditions, more mature C3 is produced and secreted to the blood stream, enhancing its own replenishment and trafficking (Figure 6b). This model could explain how chronic nutritional stress such as observed in obese and diabetic patients leads to increased circulating levels of C3.

## Discussion

Nutrient availability controls the development of various adaptive strategies. Both deprivation and overload of nutrients induce distinct cellular stress responses mediated through specific signaling cascades. Owing to their dynamic nature, different PTMs of proteins are mediators of these signaling pathways. The role of PTMs such as phosphorylation, acetylation and glycosylation were intensively studied in the context of nutrient signaling in essential metabolic organs, such as the liver.^34, 35^ However, the specific roles of ubiquitylation in the adaptation to nutrient stress in the liver remain elusive. Ubiquitylation is rapid, reversible and can modify proteins in various different topologies, leading to different cellular outcomes.^14, 36^ Thus our goal was to discover new regulatory ubiquitylation-mediated mechanisms underlying nutrient responses in the liver *in vivo*. This study shows for the first time how the ubiquitin system is engaged in a nutrient-stress response by presenting a comprehensive list of proteins that are differentially modified in the livers of mice subjected to nutritional stress.

Among other candidates, ER-resident and secretory proteins were enriched upon nutrient overload as compared with fasting in the livers. ER stress is known to induce retro-translocation of secretory proteins from the ER, their ubiquitylation and ER-associated degradation (ERAD) by 26S proteasome.^37^ ERAD is one of the best-characterized mechanisms of quality control of ER-originated membrane or secreted proteins. ER stress also expands the folding capacity of ER by production of chaperones, which is required to maintain the secretory capacity of cells.^38^ Thus our proteomic screen was particularly suitable to find secretory proteins, release of which potentially was dependent on nutrient availability. For instance, among the hits, we identified a key enzyme in sterol biosynthesis, 3-hydroxy-3-methylglutaryl acetylcoenzyme-A reductase, which is known to be retro-translocated, polyubiquitylated and degraded by ERAD.^39^ Importantly, our screen led to the discovery of an unexpected role of complement C3 in nutrient signaling in the liver.

C3 is cleaved into C3a and C3b, which are the active isoforms of C3. The C3a form was shown to have a strong lipogenic activity in adipocytes, increasing glucose uptake, TAG storage and reducing lipolysis.^24^ Thus C3 also has a role in postprandial lipid metabolism.^25^ Interestingly, recent work has demonstrated that C3a circulating in the blood can stimulate insulin secretion from pancreatic *β* cells.^26^ As both lipogenesis in adipocytes and insulin secretion by *β* cells are stimulated under nutrient-rich conditions, the increased secretion of C3 from the liver upon nutrient overload may essentially contribute to the nutrient signaling and thereby energy homeostasis of the whole organism. Indeed, our data strongly suggest that trafficking and secretion of C3 is dynamically regulated in response to nutritional challenges in isolated hepatocytes and in the liver in mice. Strikingly, we also provide the evidence that a chronic nutrient overload in obese insulin-resistant mice correlated with enhanced processing and secretion of C3. These findings go in line with published data showing that blood C3 levels are higher in obese and diabetic patients, possibly contributing to the development of low-grade inflammation and insulin resistance,^28^ as it is known that obesity-linked inflammation mediates insulin resistance.^40^ Several studies have shown that increased blood C3 levels are associated with the level of obesity, being considered a risk factor to the development of diabetes and cardiovascular diseases.^27^

Taken together, our data support a model how nutrient availability controls trafficking and secretion of C3 from the liver. Low nutrient levels inhibit C3 secretion to the blood and may thereby counteract C3-mediated stimulation of insulin secretion from pancreas^26^ under these conditions. On the other hand, nutrient overload stimulates intracellular trafficking of C3 and as a consequence its secretion. It is interesting to speculate that nutrient-dependent ubiquitylation of C3 may have an active role in this process and might regulate intracellular C3 cleavage, as proposed by a recent study reporting intracellular cleavage and activation of C3 into C3a and C3b.^41^ Our data, together with a large body of published evidence, rather supports a model in which nutrient overload induces ER stress and expands the secretory capacity of hepatocytes leading to increased ubiquitylation and degradation of a misfolded fraction of secretory proteins. Indeed, our data show that chronic nutrient overload induced an ER-stress response in mice with increased C3 secretion. Thus nutrient-dependent regulation of complement C3 secretion might be part of an evolutionary ancient innate immune mechanism to protect against pathogens that may have entered our body with food. In a chronic overnutrition state, this may contribute to persistent low-grade inflammation, a hallmark of obesity that is known to trigger insulin resistance. In future, it will be important to understand the role of nutrient-induced ubiquitylation of other secretory proteins identified in our study and the underlying molecular mechanisms.

## Materials and Methods

### Cell culture

HEK293T cells were maintained in DMEM with 10% FCS and transfected with JetPEI (Polyplus Transfection, Strasbourg, France) following the manufacturer's protocol. Primary hepatocytes were isolated as previously reported^42^ and transfected with Lipofectamine 2000 (Invitrogen, Carlsbad, CA, USA) according to the manufacturer's instructions. For experiments using different media in cells: growing culture, DMEM 1 g/l glucose, 10% FCS, 0.1 *μ*M insulin, 1 mM glutamine, penicillin–streptomycin; starvation medium, DMEM 1 g/l glucose, penicillin–streptomycin; rich medium, DMEM 4.5 g/l glucose, 10% FCS, 0.1 *μ*M insulin, 1 mM glutamine, penicillin–streptomycin.

For I*κ*B ubiquitylation experiments, cells were treated with 10 ng/ml TNF*α* and 20 *μ*M MG132 for 3 h. For biochemical validation experiments, cells were treated with 10 *μ*M MG132 for 4 h, 10 *μ*M PR619 (LifeSensors, Malvern, PA, USA) for 2 h and, when indicated, the media was replaced with HBSS for 4 h.

### Fasting and refeeding of mice

Eight–10 weeks of age C57BL/6J wild-type mice (Charles River, Saint-Germain-Nuelles, France) were used for experiments. Mice were fasted for 16 h and either killed at 1000 hours or refed a high-sucrose diet (D00041102; Research Diets Inc., New Brunswick, NJ, USA) for 24 h and killed the next day at 1000 hours. Livers were homogenized in ice-cold lysis buffer and stored at −80 °C until use. C3 knockout mice and *ob/ob* BTBR mice were obtained from Jackson Laboratory (l'Arbresel, France).

### Protein immunoprecipitation and pulldown

For the proteomic screen, ubiquitylated proteins were pulled-down with TUBEs 1 (LifeSensors, Malvern, PA, USA) and UbiQapture (Enzo Life Sciences, France) according to the manufacturer's instructions.

FLAG-tagged proteins were immunoprecipitated with FLAG M2 affinity gel (Sigma Aldrich, St Louis, MO, USA) and eluted two times with lysis buffer+0.05 mg of FLAG peptide at 4 °C. I*κ*B was immunoprecipitated using anti-I*κ*B conjugated to agarose beads (CST, Danvers, MA, USA). Tandem affinity purification of ubiquitylated proteins in denaturing conditions (8 M urea) were performed as described in Meierhofer *et al.*^43^

The following antibodies were used: anti-ubiquitin (P4D1; Santa Cruz Biotechnology, Dallas, TX, USA), anti-I*κ*B (CST), anti-GAPDH (G9545; Sigma Aldrich), streptavidin-HRP (Thermo Fisher Scientific, Waltham, MA, USA), anti-C3a (BD), and anti-p-IRE1*α* (Thermo Fisher Scientific).

### Protein digestion and nano-LC-MS/MS analysis of proteins

In-gel digestion was performed overnight at 37 °C. For in-solution digestions, proteins were solubilized in urea lysis buffer and digested with trypsin (Promega, Madison, WI, USA). Peptides were desalted on C18 reverse phase (The Nest Group Inc., Southborough, MA, USA) according to the manufacturer's protocol. MS measurements were performed on a LTQ Orbitrap XL mass spectrometer (Thermo Fisher Scientific).^44^

### Data processing

Acquired spectra were processed with Maxquant (version 1.2.2.3)^45^ against the canonical mouse proteome reference data set (http://www.uniprot.org). The search parameters were set to include only fully tryptic peptides (KR/P) containing up to two missed cleavages. Carbamidomethyl (+57.021465 amu) on Cys was set as static peptide modification. Oxidation (+15.99492 amu) on Met and ubiquitinylation (+114.042927 amu) on Lys were set as dynamic peptide modifications. The precursor mass tolerance was set to 10 p.p.m., the fragment mass error tolerance to 0.5 Da. The false discovery rate was set to 1.0% for both peptide and protein identifications; the minimum peptide length was set to 5.

One-way ANOVA was performed with the software Perseus (Martinsried, Germany)^45, 46^ to identify differentially ubiquitylated proteins, which were selected based on significance (*P*>0.05; −log *P*-value >1.3) and fold change. All identified proteins or the fasted–refed-specific proteins were submitted to Database for Annotation, Visualization and Integrated Discovery (DAVID)^47, 48^ and to Reactome^49^ online tools.

### C3 ELISA

C3 levels in the supernatant of plated primary hepatocytes and in serum of mice were measured using the Complement C3 Mouse ELISA Kit (ab157711, Abcam, Cambridge, UK) according to the manufacturer's instructions.

### Immunofluorescence

After fasting and refeeding of mice, the livers were fixed by PFA 4% in PBS. Cryosections were incubated at 4 °C with the following antibodies: anti-C3 (BS4871, Bioss Inc., Woburn, MA, USA), anti-LAMP2 (ab13524, Abcam), anti-Calnexin (sc-6465, Santa Cruz), and anti-Giantin (ab37266, Abcam). Images were taken using confocal microscope (Leica Spinning Disk Andor/Yokogawa, Belfast, UK).

### Membrane fractionation

After fasting and refeeding, the livers of mice were dissected, freshly homogenized and subjected to sequential differential centrifugation according to Ge *et al.*^50^

### Statistical analysis

Student's *t*-test was used to compare two groups, unless stated otherwise. To identify whether overlaps between ≥2 groups of proteins were random, we used the hypergeometric distribution, with assumption of the mouse proteome of 40 000 proteins. For all the tests, statistical significance was considered when *P*<0.05.

## Abbreviations

BFA: Brefeldin A
ER: endoplasmic reticulum
ERAD: ER-associated degradation
JNK: c-jun N-terminal kinase
PTM: posttranslational modification
TNF*α*: tumor necrosis factor *α*
