# Supplementary material for: Liver ubiquitome uncovers nutrient-stress-mediated trafficking and secretion of complement C3
Source: Cell Death Dis. 2016 Oct 13;7(10):e2411–. doi: 10.1038/cddis.2016.312 (PMC5133979; doi:10.1038/cddis.2016.312)
Supplement: Supplementary Supplementary Figure and Table Legends [file cddis2016312x1.docx]

**Supplementary Fig S1. Transcriptional analysis of metabolic genes from livers of fasted and refed mice.**

qPCR analysis depicts relative expression of proteins involved in (A) lipid biosynthesis, (B) glycolysis, (C) gluconeogenesis, and (D) beta-oxidation in the livers of fasted and refed mice. Data are presented as means ± SDV of 2 individual experiments with mRNAs from 5 different livers each. ***p* < 0.01.

**Supplementary Fig S2. TUBEs 1 and UbiQapture efficiently purify ubiquitylated proteins.**

A, B Western blot after TUBEs 1 (A) and UbiQapture (B) pulldowns of whole-cell lysates from HEK293T cells. Cells were treated with DMSO or 10 ng/mL TNFα and 20 μM MG132 for 3 hours as indicated. Signals with an antibody against IκBα or ubiquitin (P4D1 clone) are shown separately. Arrows indicate polyubiquitylated IkB in the eluted fractions.

I, input; FT, flow through; W, wash; E, elution from 10 μL UbiQapture; E1, elution from 10 μL TUBEs 1; E2, elution from 20 μL TUBEs 1; E3, elution from 30 μL TUBEs 1; A1, elution from 10 μL agarose; A2, elution from 20 μL agarose; A3, elution from 30 μL agarose.

**Supplementary Fig S3. Putative ubiquitylated proteins identified in livers of mice, involved in metabolic pathways.**

A, B Venn diagrams comparing proteins identified in between different experiments. (A) 50 % of the TUBEs 1-identified proteins were identified in at least 2 independent experiments. (B) 73 % of the UbiQapture-identified proteins were identified in 2 independent experiments. ***p* < 0.01.

C Venn diagram comparing proteins identified with different purification techniques. 22 % of the total number of identified proteins overlapped between TUBEs 1 and UbiQapture purified proteins. ***p* < 0.01.

D Scheme of biochemical validation designed to confirm ubiquitylation of seven selected proteins. The four proteins selected from fasted mice are depicted in orange, and the three from refed mice, in green.

**Supplementary Fig S4. Starch-binding protein 1 (STBD1) is ubiquitylated in HEK293T cells and in primary hepatocytes.**

A, B Western blot after anti-FLAG immunoprecipitation (FLAG IP) (A) and Histidin-Biotin pulldowns (His-Bio pulldown) (B) of whole-cell lysates from HEK293T cells ectopically expressing FLAG-tagged STBD1 and His-Bio tagged ubiquitin. Cells expressing FLAG alone were used as a control. Cells were treated with DMSO, 10 μM of proteasome inhibitor MG132 for 4 hours or 10 μM of deubiquitinase inhibitor PR619 for 2 hours as indicated. Input, Flow-through (FT), FLAG IP or HIS-Bio pulldowns are shown separately. Signals with an antibody against FLAG-tag or revealed by streptavidin conjugated to horseradish peroxidase (Strep-HRP) are shown separately.

C Western blot after His-Bio pulldown of whole-cell lysates from primary murine hepatocytes ectopically expressing FLAG-tagged STBD1 and His-Bio tagged ubiquitin. Cells expressing FLAG alone were used as a control. Cells were treated with DMSO, MG132 or PR619 as indicated and subjected to a starvation media (SM) or to a medium rich in nutrients (RM). Input and HIS-Bio pulldowns are shown separately. Signals with an antibody against FLAG tag or revealed by streptavidin conjugated to horseradish peroxidase (Strep-HRP) are shown separately.

**Supplementary Fig S5. Pdz-domain containing protein 1 (PDZK1) is ubiquitylated in HEK293T cells and in primary hepatocytes.**

A, B Western blot after anti-FLAG immunoprecipitation (FLAG IP) (A) and Histidin-Biotin pulldowns (His-Bio pulldown) (B) of whole-cell lysates from HEK293T cells ectopically expressing FLAG-tagged PDZK1 and His-Bio tagged ubiquitin. Cells expressing FLAG alone were used as a control. Cells were treated with DMSO, 10 μM of proteasome inhibitor MG132 for 4 hours or 10 μM of deubiquitinase inhibitor PR619 for 2 hours as indicated. Input, Flow-through (FT), FLAG IP or HIS-Bio pulldowns are shown separately. Signals with an antibody against Flag tag or revealed by streptavidin conjugated to horseradish peroxidase (Strep-HRP) are shown separately.

C Western blot after His-Bio pulldown of whole-cell lysates from primary murine hepatocytes ectopically expressing FLAG-tagged PDZK1 and His-Bio tagged ubiquitin. Cells expressing FLAG alone were used as a control. Cells were treated with DMSO, MG132 or PR619 as indicated. Input and HIS-Bio pulldowns are shown separately. Signals with an antibody against FLAG-tag or revealed by streptavidin conjugated to horseradish peroxidase (Strep-HRP) are shown separately.

**Supplementary Fig S6. LIPIN1 is ubiquitylated in HEK293T cells**

A, B Western blot after anti-FLAG immunoprecipitation (FLAG IP) (A) and Histidin-Biotin pulldowns (His-Bio pulldown) (B) of whole-cell lysates from HEK293T cells ectopically expressing FLAG-tagged LIPIN1 and His-Bio tagged ubiquitin. Cells expressing FLAG alone were used as a control. Cells were treated with DMSO, 10 μM of proteasome inhibitor MG132 for 4 hours or 10 μM of deubiquitinase inhibitor PR619 for 2 hours as indicated and kept under growing culture conditions (GC) or subjected to a starvation media (Hank’s balanced salt solution, HBSS). Input, Flow-through (FT), FLAG IP or HIS-Bio pulldowns are shown separately. Signals with an antibody against Flag tag or revealed by streptavidin conjugated to horseradish peroxidase (Strep-HRP) are shown separately.

C Western blot after His-Bio pulldown of whole-cell lysates from primary murine hepatocytes ectopically expressing FLAG-tagged LIPIN1 and His-Bio tagged ubiquitin. Cells expressing FLAG alone were used as a control. Cells were treated with DMSO, MG132 or PR619 as indicated and subjected to a starvation media (SM) or kept in growing culture conditions (GC). Input and HIS-Bio pulldowns are shown separately. Signals with an antibody against FLAG-tag or revealed by streptavidin conjugated to horseradish peroxidase (Strep-HRP) are shown separately.

**Supplementary Fig S7. Pyruvate dehydrogenase kinase isosyme 4 (PDK4) is ubiquitylated in HEK293T cells.**

A, B Western blot after anti-FLAG immunoprecipitation (FLAG IP) (A) and Histidin-Biotin pulldowns (His-Bio pulldown) (B) of whole-cell lysates from HEK293T cells ectopically expressing FLAG-tagged PDK4 and His-Bio tagged ubiquitin. Cells expressing FLAG alone were used as a control. Cells were treated with DMSO, 10 μM of proteasome inhibitor MG132 for 4 hours or 10 μM of deubiquitinase inhibitor PR619 for 2 hours as indicated and kept under growing culture conditions (GC) or subjected to a starvation media (Hank’s balanced salt solution, HBSS). Input, Flow-through (FT), FLAG IP or HIS-Bio pulldowns are shown separately. Signals with an antibody against Flag tag or revealed by streptavidin conjugated to horseradish peroxidase (Strep-HRP) are shown separately.

**Supplementary Fig S8. Pyruvate dehydrogenase complex component X (PDHX) is ubiquitylated in HEK293T cells.**

A, B Western blot after anti-FLAG immunoprecipitation (FLAG IP) (A) and Histidin-Biotin pulldowns (His-Bio pulldown) (B) of whole-cell lysates from HEK293T cells ectopically expressing FLAG-tagged PDHX and His-Bio tagged ubiquitin. Cells expressing FLAG alone were used as a control. Cells were treated with DMSO, 10 μM of proteasome inhibitor MG132 for 4 hours or 10 μM of deubiquitinase inhibitor PR619 for 2 hours as indicated. Input, Flow-through (FT), FLAG IP or HIS-Bio pulldowns are shown separately. Signals with an antibody against Flag tag or revealed by streptavidin conjugated to horseradish peroxidase (Strep-HRP) are shown separately.

C Western blot after His-Bio pulldown of whole-cell lysates from primary murine hepatocytes ectopically expressing FLAG-tagged PDHX and His-Bio tagged ubiquitin. Cells expressing FLAG alone were used as a control. Cells were treated with DMSO, MG132 or PR619 as indicated. Input and HIS-Bio pulldowns are shown separately. Signals with an antibody against FLAG-tag or revealed by streptavidin conjugated to horseradish peroxidase (Strep-HRP) are shown separately.

**Supplementary Fig S9. Enolase 1 (ENO1) is ubiquitylated in HEK293T cells and in primary hepatocytes.**

A, B Western blot after anti-FLAG immunoprecipitation (FLAG IP) (A) and Histidin-Biotin pulldowns (His-Bio pulldown) (B) of whole-cell lysates from HEK293T cells ectopically expressing FLAG-tagged ENO1 and His-Bio tagged ubiquitin. Cells expressing FLAG alone were used as a control. Cells were treated with DMSO, 10 μM of proteasome inhibitor MG132 for 4 hours or 10 μM of deubiquitinase inhibitor PR619 for 2 hours as indicated. Input, Flow-through (FT), FLAG IP or HIS-Bio pulldowns are shown separately. Signals with an antibody against Flag tag or revealed by streptavidin conjugated to horseradish peroxidase (Strep-HRP) are shown separately.

C Western blot after His-Bio pulldown of whole-cell lysates from primary murine hepatocytes ectopically expressing FLAG-tagged ENO1 and His-Bio tagged ubiquitin. Cells expressing FLAG alone were used as a control. Cells were treated with DMSO, MG132 or PR619 as indicated. Input and HIS-Bio pulldowns are shown separately. Signals with an antibody against FLAG tag or revealed by streptavidin conjugated to horseradish peroxidase (Strep-HRP) are shown separately.

**Supplementary Fig S10. Hormone-sensitive dehydrogenase11 beta 1 (HSD11β1) is ubiquitylated in HEK293T cells.**

Western blot after anti-FLAG immunoprecipitation (FLAG IP) of whole-cell lysates from HEK293T cells ectopically expressing FLAG-tagged HSD11Β1 and His-Bio tagged ubiquitin. Cells expressing FLAG alone were used as a control. Cells were treated with DMSO, 10 μM of proteasome inhibitor MG132 for 4 hours or 10 μM of deubiquitinase inhibitor PR619 for 2 hours as indicated and kept under growing culture conditions (GC) or subjected to a starvation media (Hank’s balanced salt solution, HBSS). Input, Flow-through (FT) and FLAG IP are shown separately. Signals with an antibody against FLAG-tag or revealed by streptavidin conjugated to horseradish peroxidase (Strep-HRP) are shown separately.

**Supplementary Fig S11. Pathway enrichment amongst all identified ubiquitylated proteins.**

A Pie chart depicting the Cellular Compartment Gene Ontology (GO) terms that represent identified proteins. The percent contribution of each cellular compartment is indicated.

B Pie chart depicting the most representative pathways groups enriched within all the identified proteins. The percent contribution of each large group of pathways is indicated.

C Bar chart with a detailed view of indicated pathways enriched amongst detected proteins, with the respective significance levels. – log10 of *p*-value is given to all detected proteins and – log2 of *p* -value is given to differentially identified proteins in fasted (orange) and refed (green) mice.

**Supplementary Fig S12. ER-stress induction in the liver of mice refed a high sucrose diet.**

A qPCR analysis depicts the relative expression of ER-stress markers. Data are presented as means ± SDV of 2 individual experiments with mRNAs from 5 different livers each. ***p* < 0.01.

B Western blot with whole-liver lysates of 4 fasted and 4 refed mice. To monitor ER stress, phosphorylation of inositol-requiring enzyme 1 alpha (p-IRE1α) was assessed. Tubulin was used as a loading control.

**Supplementary Fig S13. Refeeding of mice does not lead to changes in transcription and localization of C3.**

A, B qPCR analysis shows relative expression of C3 in (A) the livers of fasted and refed mice and in (B) primary murine hepatocytes subjected to a starvation media (SM) or to a medium rich in nutrients (RM) for 4 hours. Data are presented as means ± SDV of 2 individual experiments with mRNAs from 3 different livers each. ** *p* < 0.05.

C – E Intracellular localization of C3 in livers of fasted and refed mice shown by immunofluorescence co-stained with ER marker calnexin (C), Golgi marker giantin (D) and lysosomal marker Lamp2 (E). Nuclei were stained with DAPI.

**Supplementary Table S1**

**Supplementary Table S2**

**Supplementary Table S3**
