# Supplementary figures and images for: Liver ubiquitome uncovers nutrient-stress-mediated trafficking and secretion of complement C3
Source: Cell Death Dis. 2016 Oct 13;7(10):e2411–. doi: 10.1038/cddis.2016.312 (PMC5133979; doi:10.1038/cddis.2016.312)

A

TUBEs 1

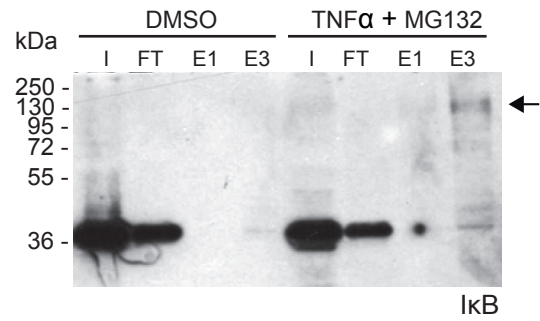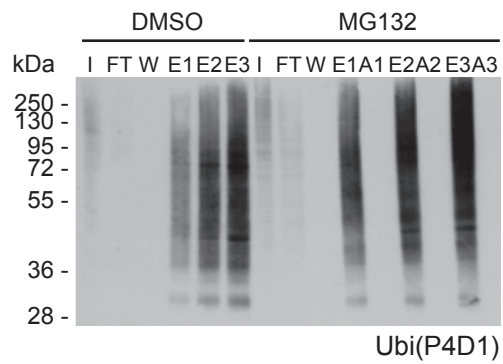

B

UbiQapture

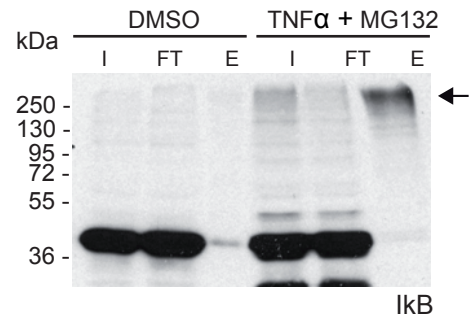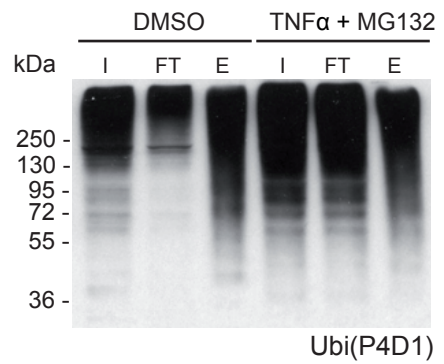

Supplement: Supplementary Figure S2 [file cddis2016312x3.pdf]

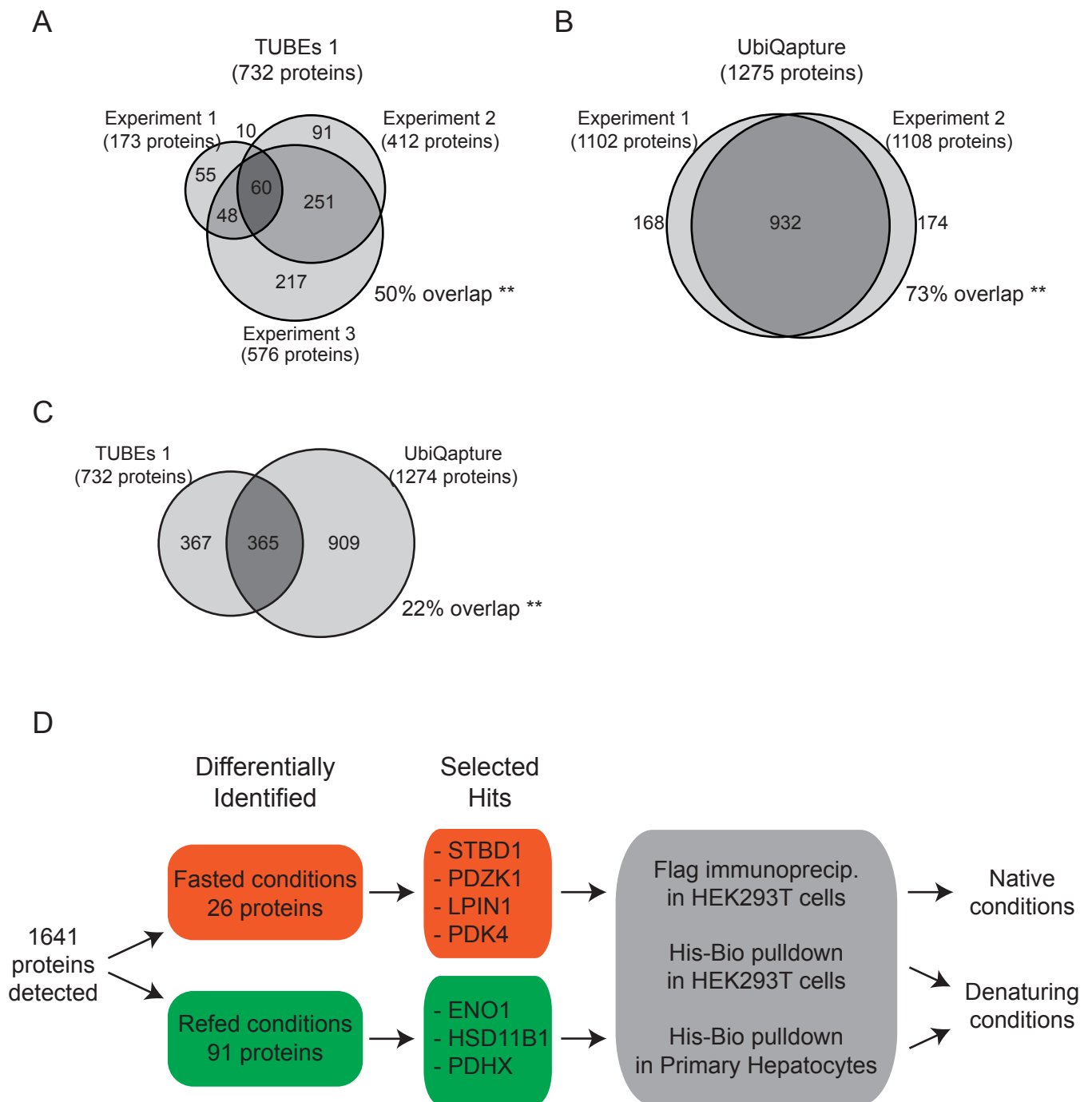

Figure S3, Magliarelli et al 2016

Supplement: Supplementary Figure S3 [file cddis2016312x4.pdf]

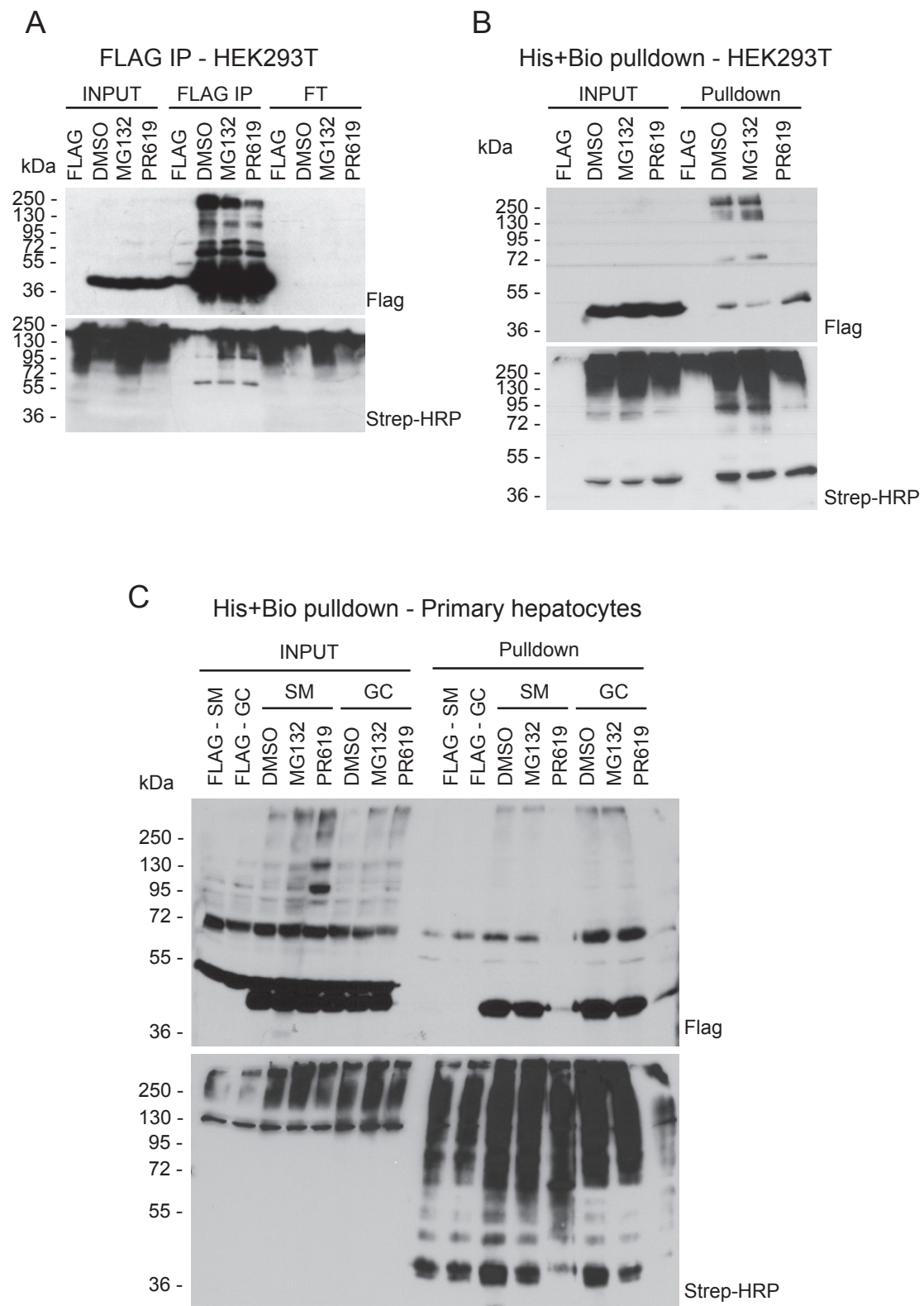

Figure S4, Magliarelli et al 2016

Supplement: Supplementary Figure S4 [file cddis2016312x5.pdf]

**A**

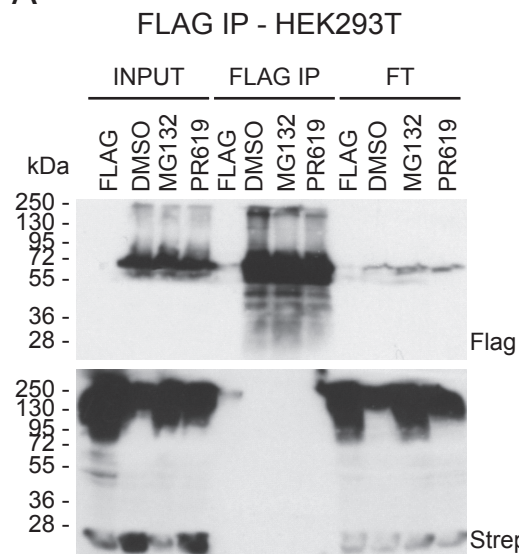

**B**

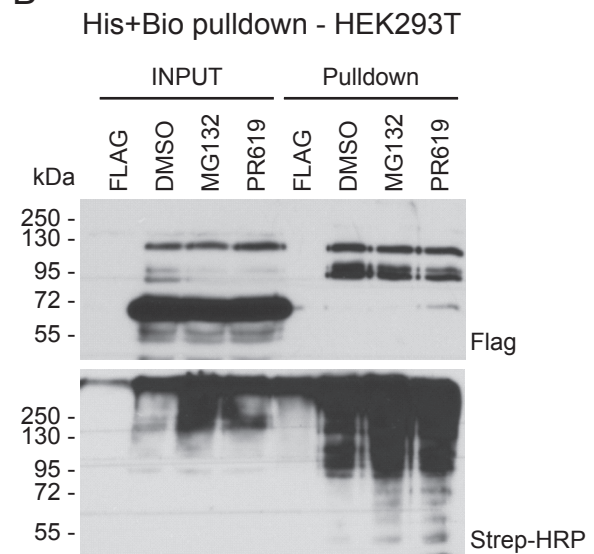

**C**

**His+Bio pulldown - Primary hepatocytes**

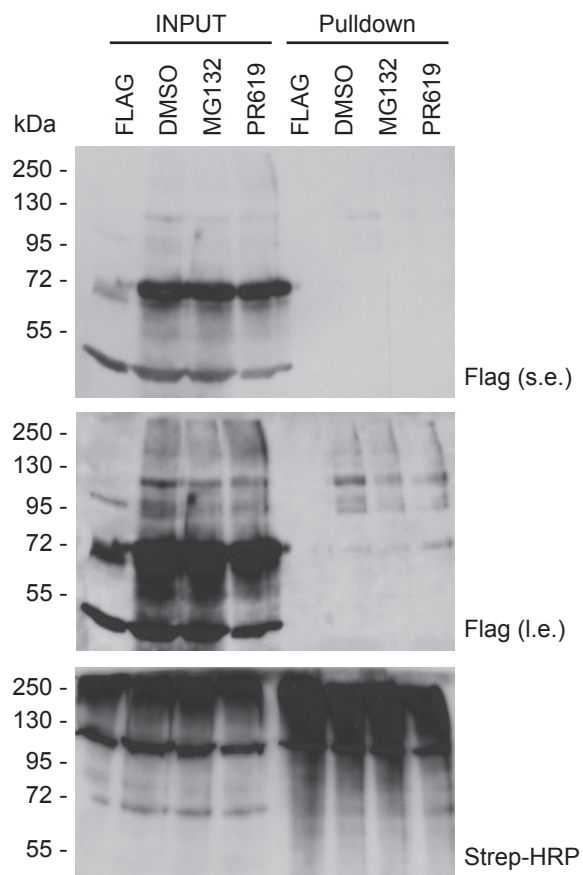

Figure S5, Magliarelli et al 2016

Supplement: Supplementary Figure S5 [file cddis2016312x6.pdf]

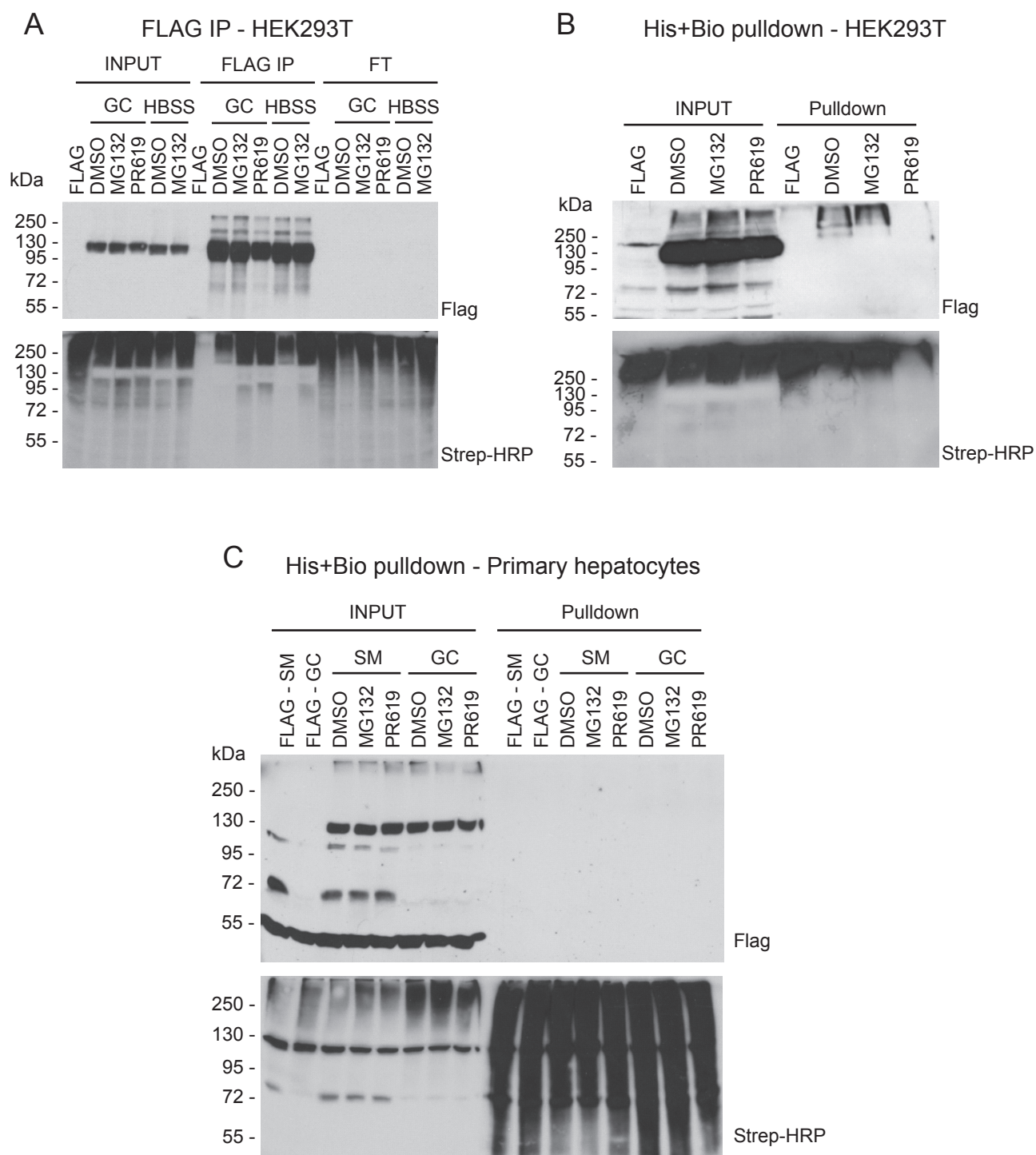

Figure S6, Magliarelli et al 2016

Supplement: Supplementary Figure S6 [file cddis2016312x7.pdf]

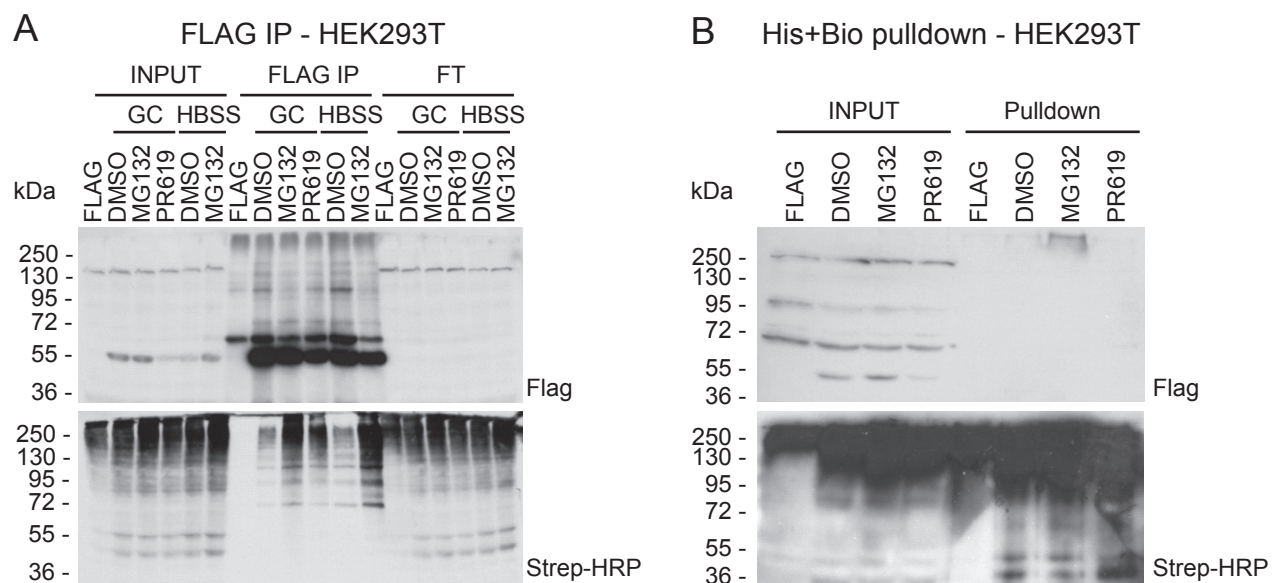

Figure S7, Magliarelli et al 2016

Supplement: Supplementary Figure S7 [file cddis2016312x8.pdf]

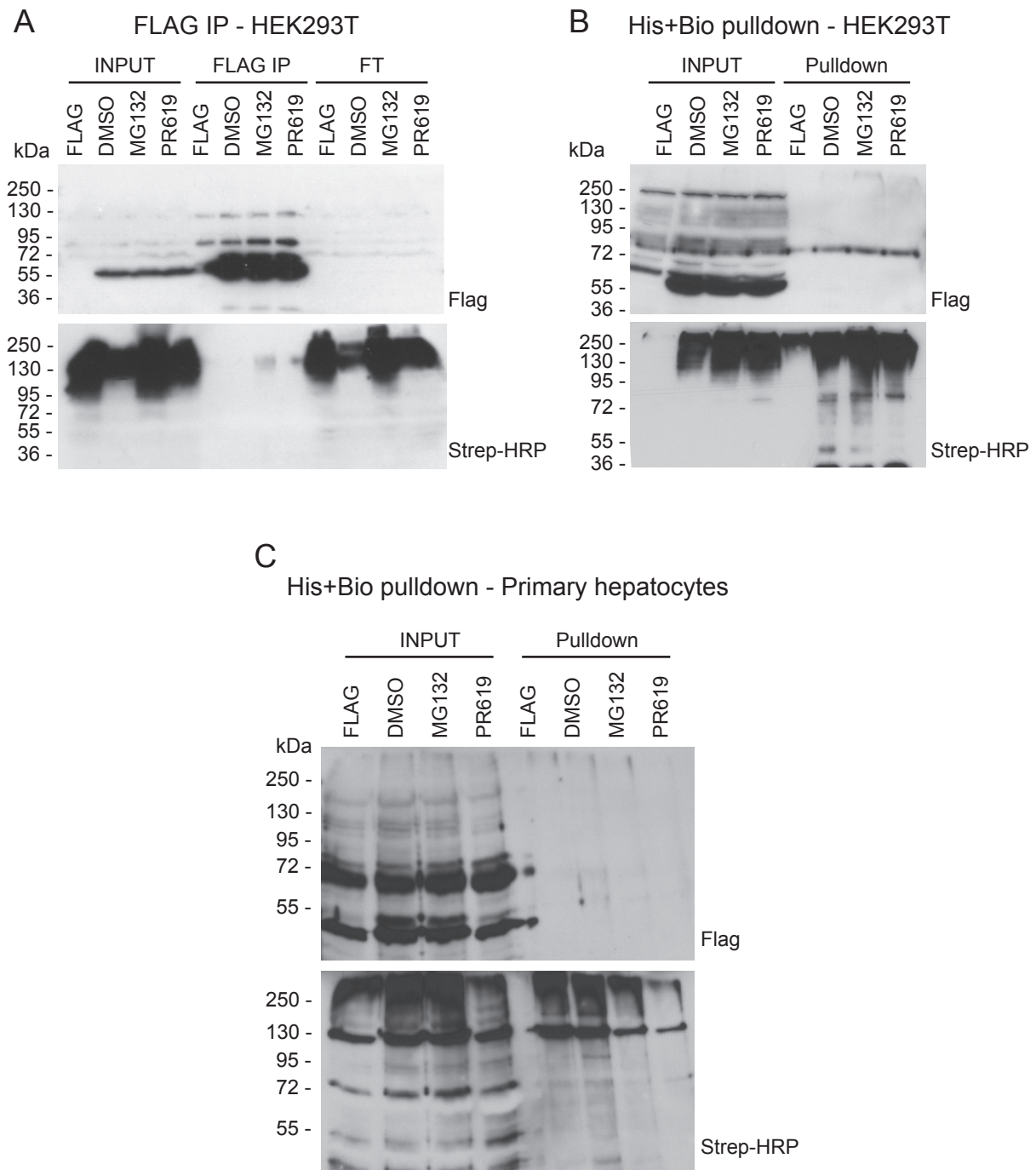

Figure S8, Magliarelli et al 2016

Supplement: Supplementary Figure S8 [file cddis2016312x9.pdf]

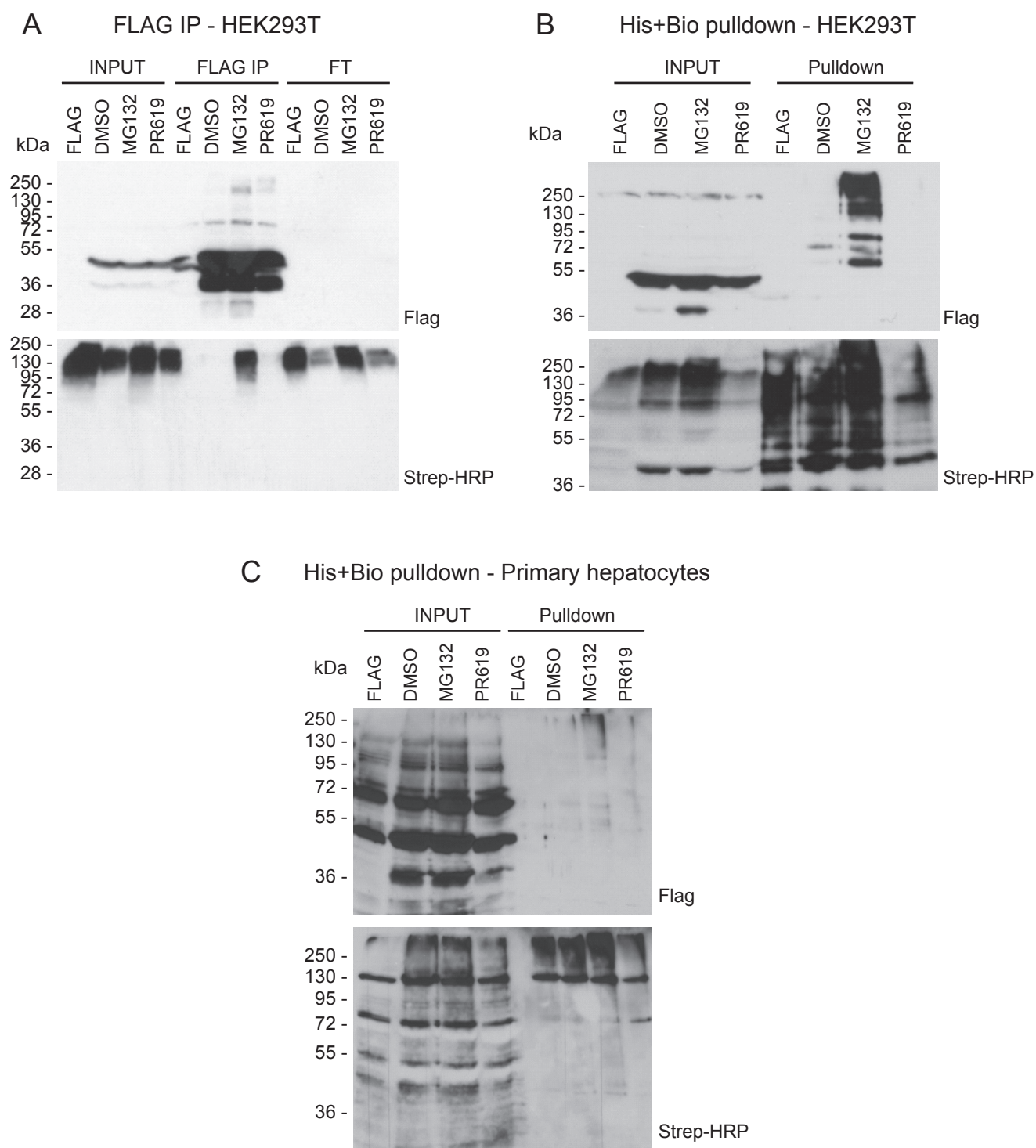

Figure S9, Magliarelli et al 2016

Supplement: Supplementary Figure S9 [file cddis2016312x10.pdf]

# FLAG IP - HEK293T

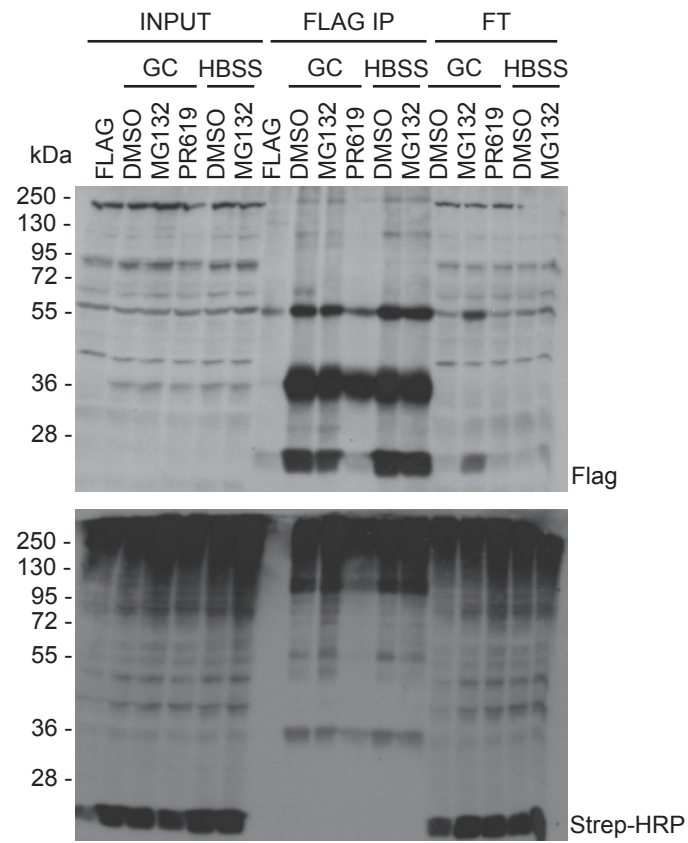

Supplement: Supplementary Figure S10 [file cddis2016312x11.pdf]

A

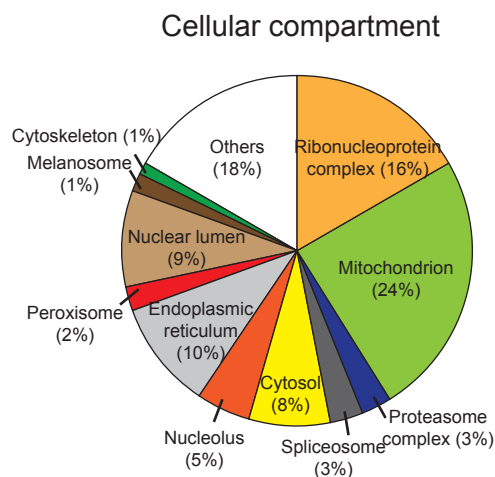

B

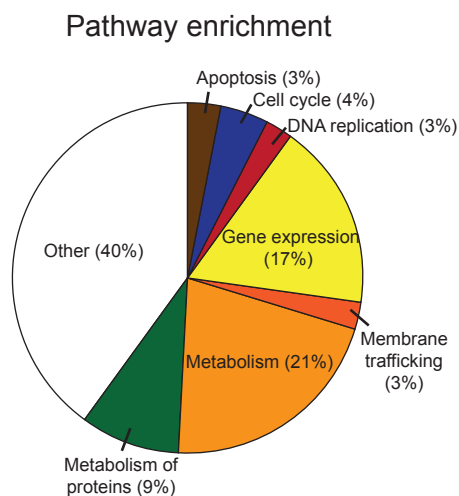

C

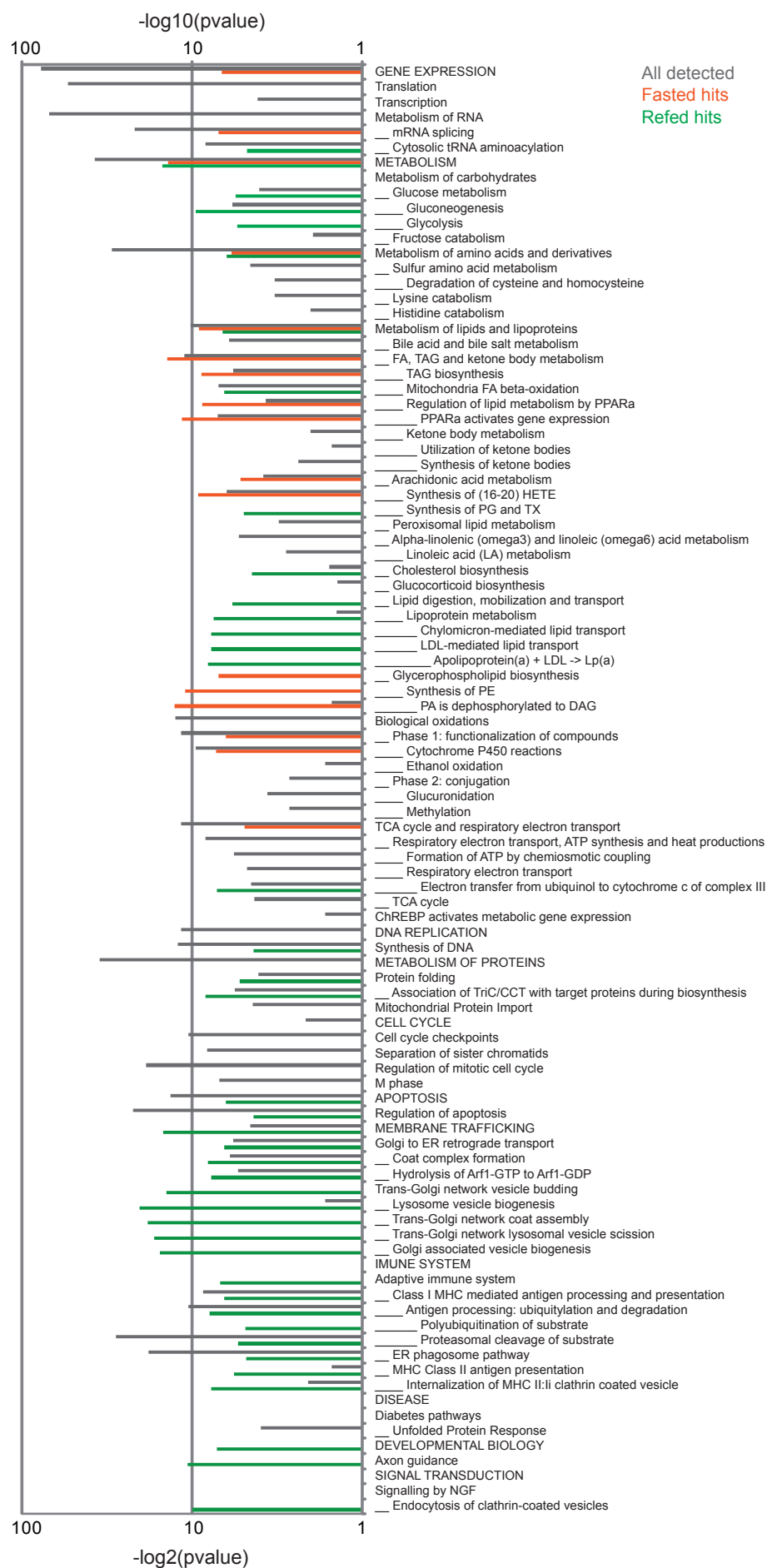

Figure S11, Magliarelli et al 2016

Supplement: Supplementary Figure S11 [file cddis2016312x12.pdf]

A

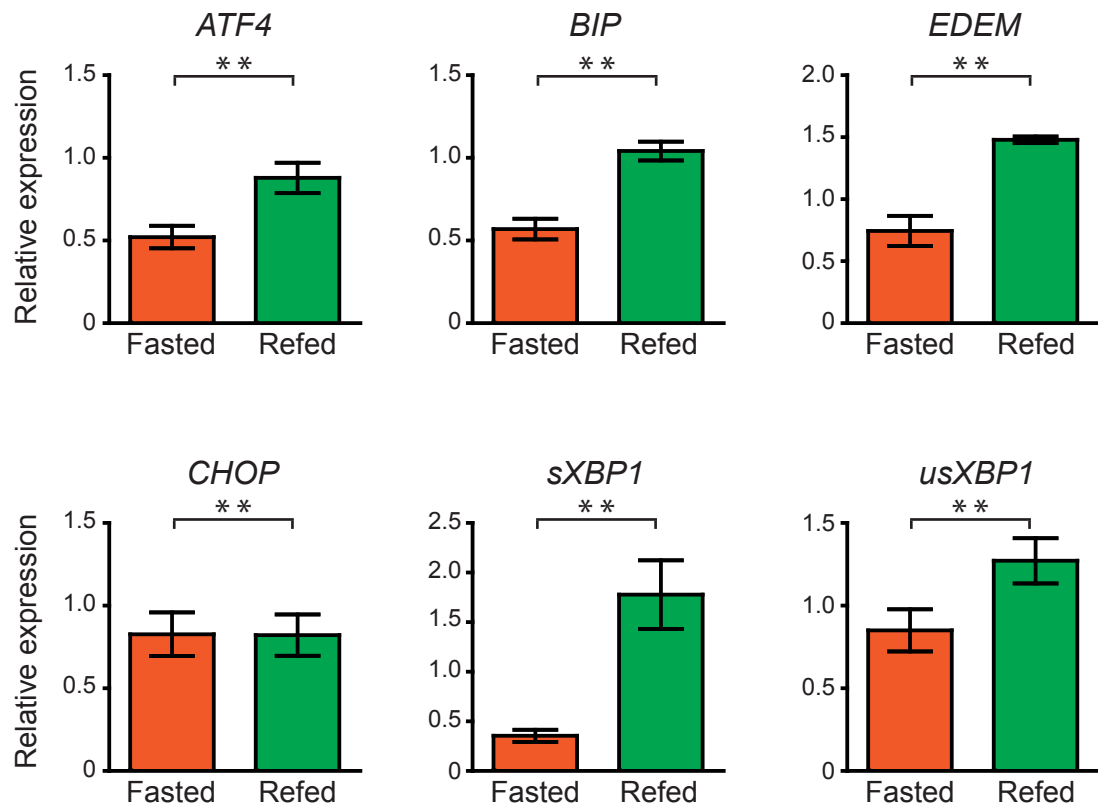

B

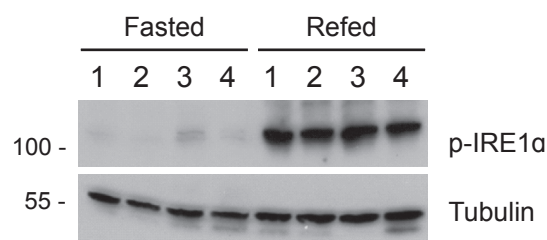

Supplement: Supplementary Figure S12 [file cddis2016312x13.pdf]

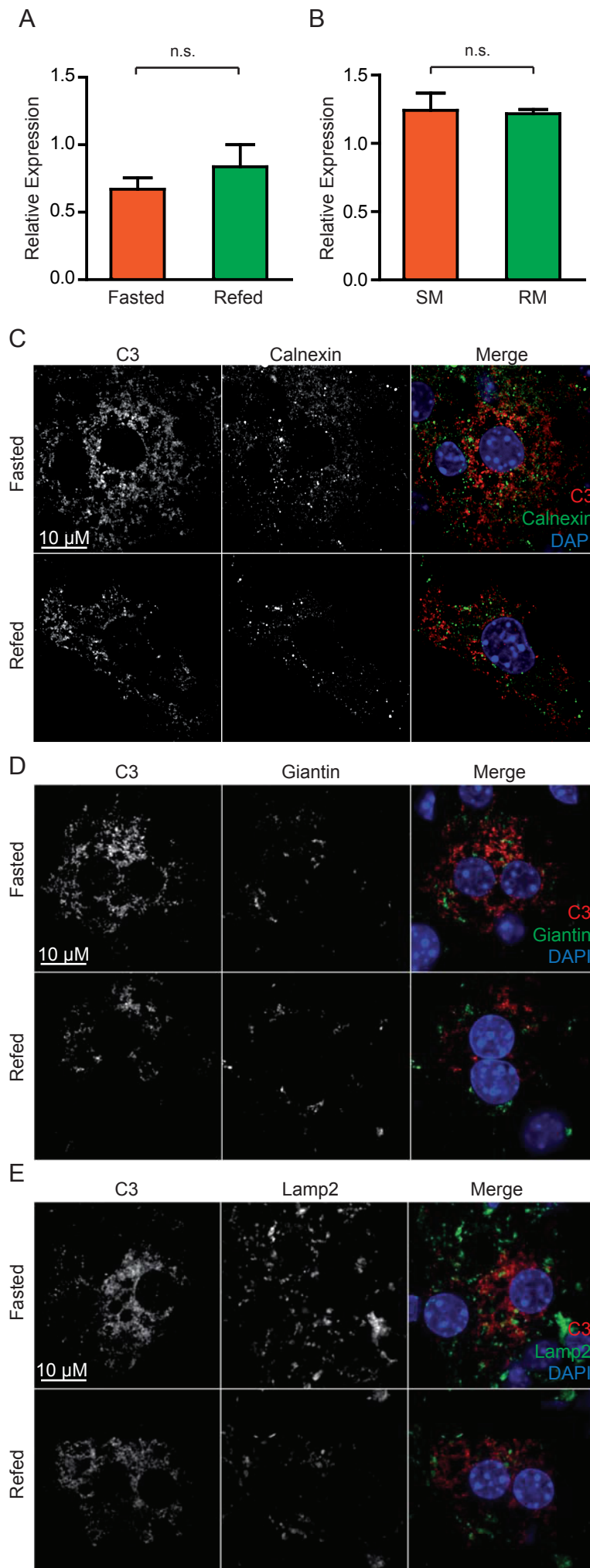

Figure S13, Magliarelli et al 2016

Supplement: Supplementary Figure S13 [file cddis2016312x14.pdf]
