# Supplementary material for: Liver ubiquitome uncovers nutrient-stress-mediated trafficking and secretion of complement C3
Source: Cell Death Dis. 2016 Oct 13;7(10):e2411–. doi: 10.1038/cddis.2016.312 (PMC5133979; doi:10.1038/cddis.2016.312)
Supplement: Supplementary Table Legends [file cddis2016312x18.docx]

**Supplementary Table 1. All proteins shown to be modified by ubiquitylation.**

Identified proteins by TUBEs1 or UbiQapture pulldowns. Table displays the method by which the protein was purified, protein names, gene names, uniprot accession numbers, protein IDs, log2 of the difference between refed and fasted and the negative log of p-value obtained by a one-way analysis of variance (ANOVA).

**Supplementary Table 2. Ubiquitylated proteins identified in livers of fasted mice.**

Setting a threshold of a 4-fold difference between fasted and refed conditions and using a p-value < 0.05, proteins differentially ubiquitylated in livers of fasted mice were selected. The table displays the method by which the protein was purified (TUBEs1 or UbiQapture), protein names, gene names and uniprot accession numbers.

**Supplementary Table 3. Ubiquitylated proteins identified in livers of refed mice.**

Setting a threshold of a 4-fold difference between fasted and refed conditions and using a p-value < 0.05, proteins differentially ubiquitylated in livers of refed mice were selected. The table displays the method by which the protein was purified (TUBEs1 or UbiQapture), protein names, gene names and uniprot accession numbers.
