# Supplementary material for: Liver ubiquitome uncovers nutrient-stress-mediated trafficking and secretion of complement C3
Source: Cell Death Dis. 2016 Oct 13;7(10):e2411–. doi: 10.1038/cddis.2016.312 (PMC5133979; doi:10.1038/cddis.2016.312)
Supplement: Supplementary Figure S1 [file cddis2016312x2.pdf]

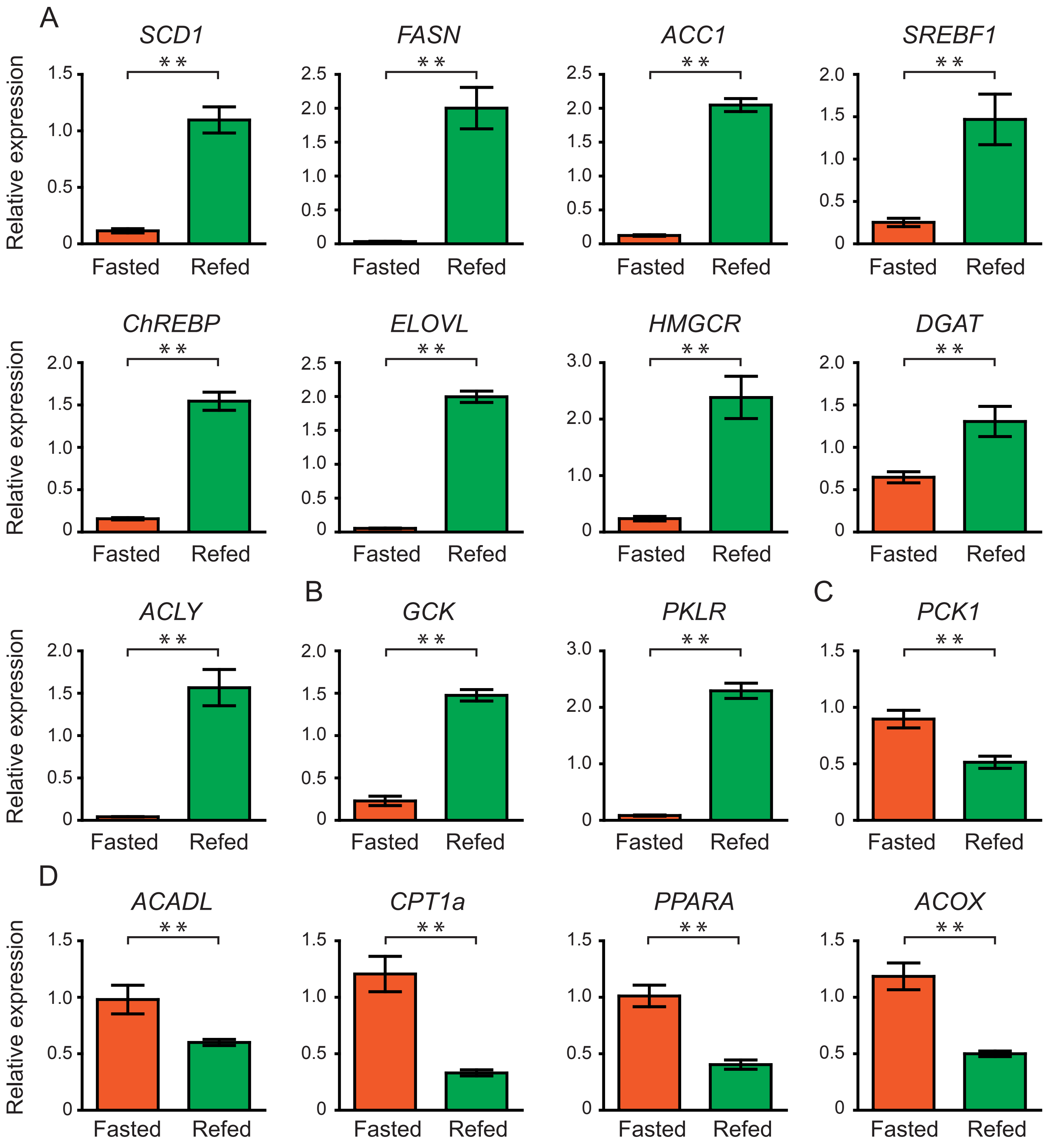

Figure S1, Magliarelli et al 2016
